# Long‐Term Earth‐Moon Evolution With High‐Level Orbit and Ocean Tide Models

**DOI:** 10.1029/2021JE006875

**Published:** 2021-12-01

**Authors:** Houraa Daher, Brian K. Arbic, James G. Williams, Joseph K. Ansong, Dale H. Boggs, Malte Müller, Michael Schindelegger, Jacqueline Austermann, Bruce D. Cornuelle, Eliana B. Crawford, Oliver B. Fringer, Harriet C. P. Lau, Simon J. Lock, Adam C. Maloof, Dimitris Menemenlis, Jerry X. Mitrovica, J. A. Mattias Green, Matthew Huber

**Affiliations:** ^1^ Department of Climate and Space Sciences and Engineering University of Michigan Ann Arbor MI USA; ^2^ Rosenstiel School for Marine and Atmospheric Science University of Miami Miami FL USA; ^3^ Department of Earth and Environmental Sciences University of Michigan Ann Arbor MI USA; ^4^ Institut des Géosciences de L'Environnement (IGE) Grenoble France; ^5^ Laboratoire des Etudes en Géophysique et Océanographie Spatiale (LEGOS) Toulouse France; ^6^ Jet Propulsion Laboratory California Institute of Technology Pasadena CA USA; ^7^ Department of Mathematics University of Ghana Accra Ghana; ^8^ Norwegian Meteorological Institute Oslo Norway; ^9^ Institute of Geodesy and Geoinformation University of Bonn Bonn Germany; ^10^ Department of Earth and Environmental Sciences Columbia University New York NY USA; ^11^ Scripps Institution of Oceanography University of California La Jolla CA USA; ^12^ Swift Navigation San Francisco CA USA; ^13^ Department of Physics Kenyon College Gambier OH USA; ^14^ Department of Civil and Environmental Engineering Stanford University Stanford CA USA; ^15^ Department of Earth and Planetary Sciences University of California Berkeley CA USA; ^16^ Department of Earth and Planetary Sciences Harvard University Cambridge MA USA; ^17^ Division of Geological and Planetary Sciences California Institute of Technology Pasadena CA USA; ^18^ Department of Geosciences Princeton University Princeton NJ USA; ^19^ School of Ocean Sciences Bangor University Menai Bridge UK; ^20^ Department of Earth, Atmospheric, and Planetary Sciences Purdue University West Lafayette IN USA

**Keywords:** ocean tides, lunar orbit, Earth‐Moon history, Earth rotation, plate tectonics

## Abstract

Tides and Earth‐Moon system evolution are coupled over geological time. Tidal energy dissipation on Earth slows Earth′s rotation rate, increases obliquity, lunar orbit semi‐major axis and eccentricity, and decreases lunar inclination. Tidal and core‐mantle boundary dissipation within the Moon decrease inclination, eccentricity and semi‐major axis. Here we integrate the Earth‐Moon system backwards for 4.5 Ga with orbital dynamics and explicit ocean tide models that are “high‐level” (i.e., not idealized). To account for uncertain plate tectonic histories, we employ Monte Carlo simulations, with tidal energy dissipation rates (normalized relative to astronomical forcing parameters) randomly selected from ocean tide simulations with modern ocean basin geometry and with 55, 116, and 252 Ma reconstructed basin paleogeometries. The normalized dissipation rates depend upon basin geometry and Earth′s rotation rate. Faster Earth rotation generally yields lower normalized dissipation rates. The Monte Carlo results provide a spread of possible early values for the Earth‐Moon system parameters. Of consequence for ocean circulation and climate, absolute (un‐normalized) ocean tidal energy dissipation rates on the early Earth may have exceeded today′s rate due to a closer Moon. Prior to ∼3Ga, evolution of inclination and eccentricity is dominated by tidal and core‐mantle boundary dissipation within the Moon, which yield high lunar orbit inclinations in the early Earth‐Moon system. A drawback for our results is that the semi‐major axis does not collapse to near‐zero values at 4.5 Ga, as indicated by most lunar formation models. Additional processes, missing from our current efforts, are discussed as topics for future investigation.

## Key Points

- Long‐term Earth‐Moon system evolution is estimated with backwards‐in‐time integrations using high‐level orbit and ocean tide models
- Rapid Earth rotation reduces paleotidal energy dissipation rate relative to paleotidal forcing. Ocean basin geometry is another key factor
- Tidal and core/mantle boundary dissipation within the Moon significantly impact the orbital evolution from about 3–4.5 Ga in the past

## 1 Introduction

At the present day, the Moon passes over the same terrestrial longitude every 24.8 hr, on average. The principal lunar semi‐diurnal tide, $M_{2}$, has half this period, 12.4 hr. The second largest diurnal tidal constituent, $O_{1}$, has a period of 25.8 hr. Energy dissipation from these tides and other semi‐diurnal and diurnal constituents, in both the ocean and the solid Earth, cause the semi‐major axis (a) of the lunar orbit around Earth to increase, and Earth′s sidereal rotation rate ($\omega_{E}$) to decrease (e.g., J. G. Williams & Boggs, 2016). The obliquity (ε) of Earth′s equator plane to the ecliptic plane, and the eccentricity (e) and inclination (i) of the lunar orbit, also change due to the action of tides on Earth. In addition, dissipation from tides within the Moon, and from coupling at the lunar core‐mantle boundary, affect the evolution of a, i, and e (e.g., J. G. Williams et al., 2001). Past and present tidal dissipation, and the related evolution of the Earth‐Moon system, are the main subjects of this study.

Our novel approach to long‐term Earth‐Moon evolution combines two numerical modeling tools. An explicit high‐level (i.e., not idealized) ocean tide model is used to simulate ocean tides over geological time, during which both Earth′s rotation rate and ocean basin geometries have undergone substantial changes. Our ocean tide model (Schindelegger et al., 2018) computes the self‐attraction and loading (SAL) term (Farrell, 1972; Hendershott, 1972), described in Section 4.1, with a method that is accurate, convenient, and computationally efficient. A “high‐level” orbital dynamics model (J. G. Williams & Boggs, 2016), which includes effects of tides and core‐mantle boundary dissipation within the Moon (J. G. Williams & Boggs, 2015; J. G. Williams et al., 2001), is used in conjunction with the ocean tide model results to simulate Earth‐Moon history. The orbital dynamics model begins at the present‐day and is run backwards over 4.5 billion years (4.5 Ga), the approximate age of the Earth‐Moon system.

The ultimate, overarching goal of this research is to link our backward Earth‐Moon system results with evolution models of the early Earth‐Moon system (e.g., Benz et al., 1986, 1987, 1989; Cameron, 1997; Cameron & Benz, 1991; Cameron & Ward, 1976; Canup & Asphaug, 2001; Ćuk et al., 2016, 2019; Hartmann & Davis, 1975; Lock et al., 2018; Melosh & Kipp, 1989, amongst many), which generally begin with a collision between Earth and a Mars‐sized body at about 4.40–4.54 Ga. A connection is made here between our predicted backwards orbital evolution trajectories and forward evolution models of the early Earth‐Moon system. Similar efforts will be advanced in future work, after additional processes are included, as discussed in Section 8.3.

Below, we introduce tidal energy dissipation, and the processes that control it on geological time scales, in more detail. We also outline the paper goals and organization.

### 1.1 Tidal Energy Dissipation

The energy dissipation of tides on Earth decreases Earth′s rotation rate and increases Earth‐Moon distance (Cartwright, 1993; Darwin, 1892; Goldreich, 1966; Hansen, 1982; Kagan & Sundermann, 1996; Kaula, 1964; MacDonald, 1964; W. Munk, 1968; Ray, 1994; Rubincam, 2016; Touma & Wisdom, 1994). Lunar laser ranging reveals that the present‐day 3.7 TW tidal energy dissipation rate (Egbert & Ray, 2003) is associated with a 3.8 cm/year increase in the geocentric lunar orbit semi‐major axis (Dickey et al., 1994; J. Müller et al., 2019; J. G. Williams & Boggs, 2016; J. G. Williams et al., 1978). Tidal dissipation also increases Earth′s obliquity ε (J. G. Williams & Boggs, 2016). Here we are referring to the mean value of obliquity, but climatically important variations in obliquity and other Earth‐Moon system parameters, as described by Milankovitch cycles, also change over geological time. The present‐day dissipation rate is dominated by tides in the ocean, with small contributions from dissipation in the solid Earth (e.g., Ray et al., 2001). However, some have argued that dissipation in the early Earth‐Moon system (the first 100 million to one billion years or so) was predominantly in the solid Earth (Ross & Schubert, 1989). Complex rotational coupling between the core and the mantle exert torques that can also affect Earth′s length of day (e.g., Correia, 2006; Greff‐Lefftz & Legros, 1999). The evolution of solid Earth dissipation and core‐mantle coupling is beyond the scope of the analysis here, but is discussed as a topic of future investigation in Section 8.3.

In discussions of tidal dissipation over geological time, it is important to distinguish between the absolute value of dissipation, and dissipation relative to astronomical forcing factors, which change over geological time due to changes in Earth‐Moon distance and other Earth‐Moon system parameters. Relative to astronomical forcing factors, present‐day ocean tidal energy dissipation rate is exceptionally high; however, the absolute (un‐normalized) present‐day dissipation rate is not necessarily exceptionally high. Later, we will define precisely “ksinχ” factors for the phase‐shifted potential from tides. Here, k is the Love number associated with the gravitational potential, and χ is a frictional phase lag. The “ksinχ” factors, commonly used in the orbital dynamics literature (e.g., J. G. Williams & Boggs, 2016), serve as proportionality agents between tidal energy dissipation rates, commonly discussed in the oceanography literature, and astronomical forcing parameters. Following J. G. Williams and Boggs (2016), we use the “ksinχ” formulation when discussing the impacts of tides on Earth on Earth‐Moon system evolution. Our discussion of the impacts of tidal dissipation within the Moon employs an alternative formulation based upon k/Q, where Q is the quality factor of a resonance (J. G. Williams et al., 2001); later we will relate the two formulations via Q=k/ksinχ.

If ksinχ factors are held constant at present‐day values, a backwards extrapolation in time yields the “Gerstenkorn event,” a collision between Earth and Moon at about 1.6 Ga (Bills & Ray, 1999; Gerstenkorn, 1955, 1967; 1969; Lambeck, 1977). If the Gerstenkorn event had taken place, the Moon would have an age of only 1.6 Ga, much less than the 4.40–4.54 Ga age of the Earth‐Moon system extrapolated from geochemical and geochronological evidence (e.g., Barboni et al., 2017; Borg et al., 2011; Kruijer & Kleine, 2017; Lock et al., 2020; Maurice et al., 2020). Therefore, the ksinχ factors must have been lower throughout most of Earth‐Moon history. We discuss potential reasons for lower ksinχ factors below. We acknowledge that ksinχ factors likely were larger than present‐day values for short intervals. Simulations by Egbert et al. (2004), Arbic, MacAyeal, et al. (2004), Arbic et al. (2008), Griffiths and Peltier (2009), Green (2010), and others suggest that tides and tidal energy dissipation rates during the recent ice ages (for which the Earth‐Moon distance was essentially equal to the present‐day value) were exceptionally large, due to a reduced shelf area associated with lower sea levels (Arbic & Garrett, 2010; Arbic et al., 2009). Though shelves dissipate most of the present‐day tidal energy, their removal counterintuitively yields a greater global tidal dissipation because open‐ocean tides become much larger than they are today. Because the recent ice ages represent a short timespan (∼several hundred thousand years) relative to the 4.5 Ga timescales considered in this paper, we do not consider the effects of ice age tides further in this paper. Similarly, we do not attempt to consider Milankovitch scale orbital variations (Levrard & Laskar, 2003; Lourens et al., 2001; Morrow et al., 2012).

Ocean tidal energy dissipation rate depends on the strengths of tidal currents, which, as with elevations, are affected by resonances in the tidal system. Present‐day open‐ocean tides are thought to be in a state of (damped) resonance, because the time scale for tides to cross ocean basins—set by basin length scales and the phase speed $\sqrt{gH}$ of shallow‐water waves—is comparable to tidal forcing periods. Here, g is gravitational acceleration and H is the resting (unperturbed) water column depth. The dependence of tidal phase speeds on H makes clear that mean sea level, shaped on long time scales by tectonic forces and the presence or absence of large ice sheets, is a critical factor in ocean tide evolution. In coastal seas, where H, tidal phase speed, and horizontal length scales are smaller than in the open‐ocean, additional resonances are possible depending on the precise geometries of the region in question. For present‐day conditions, numerous coastal locations, such as Hudson Strait, the English Channel, the Bay of Fundy, the Patagonian Shelf, and so on, resonate near the semi‐diurnal and diurnal tidal frequencies (Arbic & Garrett, 2010; Arbic et al., 2007, 2009; C. Garrett, 1972; Heath, 1981; Skiba et al., 2013; Webb, 1976; Wunsch, 1972). Arbic et al. (2009) and Arbic and Garrett (2010) modeled global tides as a system of two coupled oscillators, one (the open‐ocean) with much greater mass than the other (the coast), and demonstrated that regions of large coastal tides have a substantial back‐effect on the open‐ocean tides.

Oceanic normal modes (M. Müller, 2007, 2008a, 2008b; Platzman, 1984), also called free oscillations, provide a more sophisticated understanding of tidal resonance. As with the normal modes of a vibrating drum, oceanic normal modes represent the frequencies and spatial structures that the ocean would vibrate at and with if left to oscillate freely. If the free oscillations have periods and spatial structures that match those of the astronomical forcing relatively well, then the tidal response will be large. The horizontal length scales of tides are set by the Rossby radius of deformation $L_{d}$, given by

(1) $$L_{d}=\frac{\sqrt{gH}}{f}.$$

Here the Coriolis parameter is

(2) $$f=2\omega_{E}sin\left(\phi \right),$$

where ϕ is latitude.

### 1.2 Effects of Basin Geometries and Earth’s Rotation Rate

Oceanic normal modes, ocean tidal amplitudes, and the ksinχ factors, depend on ocean basin geometry; seafloor bathymetry, continental configuration, shoreline roughness, and hypsometry. The present‐day (PD) continental configuration and value of mean sea level yields anomalously large tides, in both open‐ocean and coastal areas. At times in the past when paleogeometries were not well‐placed for resonance, tides would have been substantially smaller than they are today. For instance, during the era of the Pangean supercontinent, ocean basins were much larger and the basin traverse times and tidal periods were not as well‐matched as they are today. Green and Huber (2013) found that global ocean tidal dissipation rates in the relatively well‐constrained 55 Ma paleogeometry are only about one half the present‐day value. They also found that the narrower Atlantic was not as conducive to tidal resonance as it is in the present‐day. Green et al. (2017) explored a range of continental configurations over the past 252 Ma, and found that global tidal energy dissipation rates were generally far below the present levels, consistent with Ooe's (1989) study of the $M_{2}$ tidal constituent in the Permian ocean, and with results from Poliakow (2004). However, because plate tectonics drives a continuous opening and closing of ocean basins (Wilson, 1966), basin geometries resembling the present‐day geometry likely existed in the past. In the Phanerozoic, ocean basins have come and gone on a timescale of about 100–250 million years (Boulila et al., 2018; Nance & Murphy, 2013; Zaffos et al., 2017), much less than the 4.40–4.54 Ga age of the Earth‐Moon system, although the recurrence intervals may have varied further back in time (Brown et al., 2020; Evans, 2013; Piper, 2018). Because of its cyclical nature, basin geometry is unlikely to be the sole reason behind the lower ksinχ factors over most of the Earth‐Moon history (Bills & Ray, 1999).

Another mechanism for lower ksinχ factors in the geological past is tied to Earth′s rotation rate. G. E. Williams (2000) inferred from tidal rhythmite sediments that in the late Neoproterozoic (640 Ma), the length of day was 21.9 ± 0.4 hr. Using idealized ocean tide and orbital models, Hansen (1982) argued that shortly after the formation of the Moon at ∼4.5Ga, the length of the sidereal day was between 12 to 18 hr long. Using a different model, MacDonald (1966) determined from dynamical considerations a slightly different Earth rotation period between 9.9 and 13.1 hr just after the lunar formation. Hence, tidal horizontal length scales would have been shorter due to higher astronomical tidal forcing frequencies and the smaller Rossby radius resulting from a larger Coriolis parameter (Equations 1 and 2 with a larger value of $\omega_{E}$). Bills and Ray (1999), averaging model output over multiple geometries, found lower tides and tidal energy dissipation rates in the geologic past because of a mismatch between the spatial scales of tidal forcing and oceanic normal modes having frequencies close to tidal frequencies; see also C. J. R. Garrett and Munk (1971). The reduction in the strength of tides with faster Earth rotation rates also was predicted in the idealized ocean tide model results of Hansen (1982), Webb (1982), and Kagan and Maslova (1994), and, more recently, in simulations of exoplanet tides by Blackledge et al. (2020).

### 1.3 Paper Goals

We examine the history of the Earth‐Moon system over an assumed age of 4.5 Ga. We build upon a literature of Earth‐Moon system modeling that tends to focus either on highly idealized approaches over the full time interval or more sophisticated approaches within narrow time slices. Here we employ two “high‐level” (i.e., not idealized) tools that have, to our knowledge, not been used simultaneously in the study of long‐term Earth‐Moon evolution.

First, we use a state‐of‐the‐art ocean tide model with four different realistic basin geometries: the PD geometry, and reconstructed paleogeometries from three distant epochs. The 55 million year (Ma) geometry is relatively well‐constrained yet sufficiently different from the PD geometry that modeled 55 Ma tides depart significantly from modern tides (Green & Huber, 2013; Green et al., 2017). We also use paleogeometries from 116 Ma and 252 Ma reconstructions. The 252 Ma geometry is from the final amalgamation of the Pangean supercontinent and is therefore quite different from the other three geometries. We explore ocean tidal energy dissipation rate as a function of ocean geometry and Earth's rotation rate $\omega_{E}$.

Second, we use more sophisticated orbital evolution models than those used in some previous studies. Kagan and Sundermann (1996), for instance, use orbital evolution models that account only for the dissipation of tides on Earth (i.e., not including tidal dissipation in the Moon), and that only calculate changes to $\omega_{E}$ and a. Our evolution model also accounts for changes in ε, i, and e due to tides on Earth, and additionally includes the effects of tides and the core‐mantle boundary within the Moon (J. G. Williams et al., 2001). The latter can counteract the effects of ocean tides and solid Earth tides on a, i, and e, especially during orbital evolution from 3 to 4.5 Ga. We compute the trajectories of the Earth‐Moon system parameters $\omega_{E}$, ε, a, e, and i with all four ocean basin geometries considered separately. In addition, we employ 1,000 Monte Carlo simulations that randomly select, for each value of Earth rotation rate considered, from results employing the four geometries. The Monte Carlo simulations roughly account for uncertainties in continental positions throughout geological time. A contrasting approach is taken by Waltham (2015), who uses a much simpler orbital dynamics model constrained by known present‐day Earth‐Moon system parameters and the near‐zero value of a at 4.5 Ga to model the history of a, $\omega_{E}$, ε, and Milankovitch cycles. Another recent effort, Tyler (2021), parameterizes the effects of continental configuration in a simple Earth‐Moon system history model that fits inferences from geological proxies.

To simplify terminology, hereafter we will generally refer to “solid Earth tides” as simply “Earth tides.” When referring to their combined effects, solid Earth tides and ocean tides will often be referred to as “terrestrial tides” or “tides on Earth.” The combined effects of tides and core‐mantle boundary effects within the Moon will at times be referred to as “Moon tides.”

The discussion section of the paper establishes connections to several research threads in the literature. We compute the vertical component of the angular momentum of the Earth‐Moon system, which Tian and Wisdom (2020) argue is a strong constraint on lunar formation and Earth‐Moon system history. We compare our orbital model results with geological proxy results from tidal rhythmites. We compare our results near ∼4Ga with results from models of the early Earth‐Moon system (Ćuk et al., 2016, 2019). We compute the torques of tides raised on Earth by the Moon. Following Zahnle and Walker (1987), Bartlett and Stevenson (2016) argued that these torques were overcome by atmospheric tides, thus stabilizing Earth rotation rate for a long period (perhaps ∼2Ga) during the Precambrian. Our ocean tide model results provide a useful test of the plausibility of the atmospheric tide stabilization mechanism. We discuss the evolution over geological time of three parameters that are critical for Earth′s climate: (a) ε, which controls Earth′s seasons, (b) the Milankovitch precession rate, a key parameter in the development of ice ages, and (c) ocean tidal dissipation, which is thought to exert a strong control on the oceanic meridional overturning circulation (W. Munk & Wunsch, 1998). Lower ksinχ values in the distant past do not always translate to lower ocean tidal dissipation because the Moon in the distant past was closer than it is now. Finally, we briefly discuss implications of our research for exoplanets.

### 1.4 Paper Organization

Because this paper is written with disparate audiences in mind—Earth history specialists, geophysicists, physical oceanographers, and planetary scientists—we describe the basin geometry reconstructions, ocean tide model, and Earth‐Moon orbital evolution model in some detail. After discussing the basin geometry reconstructions in Section 2, we provide the nomenclature on tidal arguments and frequencies necessary to understand the ocean tide and orbital dynamics models in Section 3. Subsequently, we detail the workings and results of the ocean tide model (Section 4), the orbital dynamics equations (Section 5), and the results and implications of our backwards integrations (Sections 6 and 7, respectively). Section 8 summarizes our work and looks forward to improvements we intend to make in future investigations.

## 2 Ocean Basin Geometry Reconstructions

The PD ocean basin geometry is taken from RTopo‐2 (Schaffer et al., 2016), a self‐consistent compilation of seafloor bathymetry, bedrock elevation, ice‐sheet topography, and ice thickness. We use seafloor bathymetry as bottom depth in the open ocean and construct water column thickness under floating ice shelves by subtracting ice thickness below mean sea level from the bedrock depth. This global 1‐min RTtopo‐2 bathymetry is mapped to the computational grid of the ocean tide model (Section 4.1) by forming cell averages and setting shallow depths above the 5‐m contour to 5 m.

Paleogeographies are poorly constrained by observations and affected by a number of processes. For instance, seafloor spreading rates control seafloor roughness, which influences damping of tides in the open ocean (Egbert & Ray, 2000, 2001, 2003). Mean sea level is controlled by tectonic forces and the presence or absence of large ice caps. Shoreline “roughness” (indentations and other fractal‐like features), which also affects tides and tidal dissipation, is controlled by glacioeustasy and numerous other geological processes. For example, the raising and lowering of sea level during glacial‐interglacial cycles promotes the development of fjord and/or fluvio‐estuarine indentation of the shoreline. In Section 8, we will consider some statistical approaches that could be applied to the estimation of paleogeographies. For the present paper, we take reconstructed paleogeographies, as they are, from other studies.

The 55, 116, and 252 Ma reconstructed paleogeographies are based upon work done in Green and Huber (2013), Herold et al. (2014), and Green et al. (2017). The paleogeographies are based upon tectonic reconstructions from R. D. Müller, Sdrolias, Gaina, and Roest (2008) for 55 Ma, Scotese and Golonka (1992) for 116 Ma, and R. D. Müller et al. (2016) for 252 Ma. The methodology used to map tectonic data into bathymetry data is outlined in Herold et al. (2014). Basin depths are computed from the age‐depth relationship derived by Stein and Stein (1992). Where oceanic crust is not available for a time slice a symmetric mid‐ocean ridge spreading was assumed, and seafloor spreading isochrons from the conjugate plate were applied. In regions where no data were available from the conjugate plate, interpolation between available isochrons and the adjacent plate margin was used (R. D. Müller, Sdrolias, Gaina, & Roest, 2008; R. D. Müller, Sdrolias, Gaina, Steinberger, & Heine, 2008). Large igneous provinces (LIPs) were reconstructed from modern LIP outlines and an estimate of the palaeo‐LIP height based on Schubert and Sandwell (1989). The depths then were modified to accommodate sediment thickness based on the age‐latitude relationship (R. D. Müller, Sdrolias, Gaina, Steinberger, & Heine, 2008) and local ocean drilling data (Herold et al., 2014). Because detailed information for the 252 Ma time slice was not available, features such as LIPs and mid‐ocean ridges were not included.

**Figure 1:**
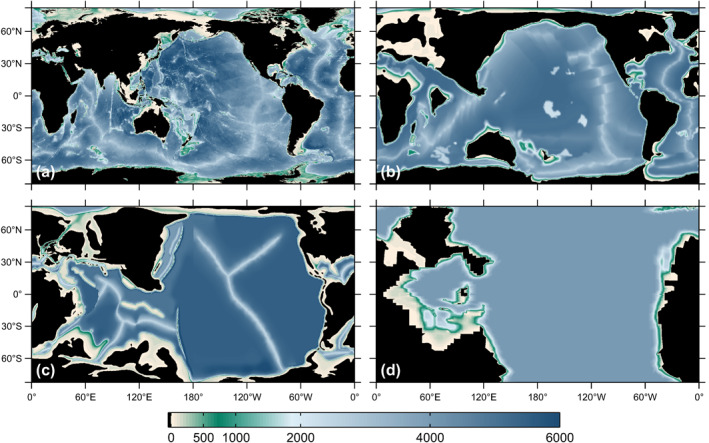
(a) Ocean basin geometry at present‐day (PD). (b–d): Reconstructed basin geometries at (b) 55 Ma, (c) 116 Ma, and (d) 252 Ma.

Maps of the PD and three deep‐time basin geometries are given in Figure 1. Aside from differences in continental locations, another clear difference between the maps is the relative smoothness of the shorelines and bathymetries in the reconstructed geometries. Green et al. (2017) showed that if the PD geometry is smoothed in a manner similar to that seen in the paleo‐reconstructions, the PD tidal dissipation increases by about 60%, suggesting therefore that tidal energy dissipation rates in simulations with smoother paleogeographies may represent over‐estimates.

## 3 Tidal Arguments and Frequencies

This section defines some of the nomenclature surrounding tidal arguments and frequencies. The nomenclature is used in both Section 4, on tide modeling, and Section 5, on the orbital evolution model. The evolution rates for orbit elements, Earth′s rotation and obliquity are given in Section 5.

The tide‐raising potential on Earth is expressed with spherical harmonic functions of degree 2; degree 3 and higher terms are small and often ignored. The degree 2 functions can be represented with a matrix in a Cartesian frame (J. G. Williams & Boggs, 2016):

(3) $$U_{ij}={\left(\frac{a}{r}\right)}^{3}u_{i}u_{j}.$$

The $u_{i}$ and $u_{j}$ are components of unit vectors from the Earth toward the Moon or Sun in a frame rotating with the Earth. The center‐to‐center distance is r. Tides on the Moon can be expressed with analogous Moon‐centered functions toward the Earth and Sun (J. G. Williams & Boggs, 2015). Combinations of the $U_{ij}$ factors, matrix elements also known as Cartesian factors, play a central role in the orbital dynamics equations of Section 5.1. Separate $U_{ij}$ functions for each tidal component ($M_{2}$, $O_{1}$, $M_{f}$, etc.) are distinguished by an additional subscript q.

The Cartesian factors define the relative amplitudes of different tidal constituents. The Cartesian factors use products of Cartesian coordinates $u_{i}u_{j}$, but they are equivalent to spherical harmonic functions for certain frequencies. In J. G. Williams and Boggs (2016), the Cartesian factors were computed using accurate orbit expansions with numerical coefficients (Chapront‐Touzé & Chapront, 1988, 1991). Because the numerical coefficients would not be constant during evolution, here we replace them with analytical expansions for an elliptical orbit. We develop Fourier series, in Section 5.1, for the Cartesian factors. The Fourier series amplitudes depend on obliquity, inclination, semi‐major axes, and eccentricities and angles that depend on Earth′s rotation and orbit mean anomaly, node, and mean longitudes of Sun and Moon.

A degree‐2 tidal potential $W_{2}$ causes a distortion of the Earth or the Moon. The potential $W_{2}$ can be expressed with the $U_{ij}$. The change in potential due to elastic deformation is given by $k_{2}W_{2}$, where $k_{2}$ is the potential Love number. When dissipation is present, we need frequency dependent Love numbers $k_{2mq}$ and phase shifts $\chi_{2mq}$. Here, $\sin\chi_{2mq}$ is equivalent to $1/Q_{tidal}$, where $Q_{tidal}$ is the “quality factor” associated with the forced‐damped tidal system. At the Earth, spherical harmonic order m is 0, 1, or 2 for long period, diurnal, and semi‐diurnal tides, respectively. At the Moon, we are interested in the potential from tides raised by Earth and the Sun. These potentials for each tidal frequency are proportional to squares of linear combinations of the $U_{ij}$ functions times $k_{2mq}\cos\chi_{2mq}$. These potentials are summed over the series of tidal terms ($M_{2}$, $K_{1}$, $O_{1}$, etc.) indicated with subscripts 2mq. The tidal response of the ocean is complicated by the shape of the oceans and other considerations described earlier, causing additional terms with various degrees and orders. For evolution, the potential response terms $V_{2mq}$ with the same degree 2, order m and tidal constituent q as each tide‐raising term are the most important for secular changes in the energy transfer and dissipation rates. Other combinations give periodic power oscillations.

Each $U_{ijq}$ has an associated orbit argument that depends on time. Part of that argument depends on θ, the sidereal rotation angle of Earth, that is, the angle between the intersection of the equatorial and ecliptic planes (equinox) and the Greenwich zero longitude. Part of the argument, $\zeta_{q}$, depends on the lunar orbit. For example, $\zeta_{0}$ = 0 is associated with the $K_{1}$ and $K_{2}$ tides and $\zeta_{1}=2L$ is associated with the $M_{2}$, $O_{1}$, and $M_{f}$ tides, where L is the Moon's mean orbit longitude. The rotation angle θ enters the argument of the potential with product mθ so that the argument of each component of $V_{2mq}$ is $m\theta +\zeta_{2mq}$ or $m\theta -\zeta_{2mq}$ for eastward and westward propagating waves, respectively. Eastward and westward refer to the direction of propagation with respect to the mean direction of the tide‐raising body (Moon or Sun). A “+” or “−” is added to the subscripts of diurnal and semi‐diurnal tides to distinguish the two directions. Long period (zonal) tides have no longitude dependence and no “+” or “−.” The eastward propagating tides are generally smaller and are ignored in our integrations of the Earth‐Moon evolution equations of Section 5.1; however, they are included in that section for the sake of completeness. The $K_{1}$ and $K_{2}$ tides do not propagate eastward or westward with respect to the mean direction of Moon or Sun.

The “Delaunay angles” are polynomial expressions for lunar mean anomaly l, solar mean anomaly $l^{\prime }$, lunar argument of latitude F, and elongation of the Moon from the Sun D. In addition, Ω is the node of the lunar orbit plane on the ecliptic plane, and lunar mean longitude L=F+Ω and solar mean longitude $L^{\prime }=L-D$ are useful; see J. G. Williams and Boggs (2016) for a more comprehensive description. Table 1 links the 14 tidal constituents considered in the orbital dynamics model of Section 5 with their tidal arguments ($m\theta -\zeta_{2mq}$ for diurnal and semi‐diurnal tides and $\zeta_{2mq}$ for long period tides), their Doodson Numbers, and their Delaunay angles. The 14 constituents include the two largest long‐period tides, the four largest diurnal tides, the four largest semi‐diurnal tides, and the diurnal and semi‐diurnal node (Ω) and L+F terms. The Ω and L+F tides, which as indicated in Table 1 differ by the nodal frequency from larger constituents, have small amplitudes but a disproportionately large influence on the lunar inclination i, as can be seen in Table 6.

**Table 1:** Values of Doodson Numbers, Delaunay Angles, Tidal Arguments, Tidal Frequencies (ω), and Integer Sets $j_{Mq}$, $j_{Wq}$, and $j_{\Omega q}$ (See Text), for the 14 Tidal Constituents Used in Our Orbital Dynamics Model^a^

Constituent	Doodson number	Delaunay angle	Tidal argument	Frequency ω	$j_{M}$	$j_{W}$	$j_{\Omega }$
Long‐period tides							
$M_{f}$	075,555	2F+2Ω	2L	2n	2	2	2
$M_{m}$	065,455	l	l	$\frac{dl}{dt}=n-\frac{d\bar{\omega }}{dt}$	1	0	0
Diurnal tides							
$K_{1}$	165,555	Constant	θ	$\omega_{E}$	0	0	0
$O_{1}$	145,555	2F+2Ω	θ−2L	$\omega_{E}-2n$	2	2	2
$P_{1}$	163,555	2F+2Ω−2D	$\theta -2L^{\prime }$	$\omega_{E}-2n^{\prime }$	0	0	0
$Q_{1}$	135,655	2F+2Ω+l	θ−2L−l	$\omega_{E}-2n-\frac{dl}{dt}=\omega_{E}-3n+\frac{d\bar{\omega }}{dt}$	3	2	2
Ω ($K_{1}$ nodal)	165,565	Ω	θ−Ω	$\omega_{E}-\frac{d\Omega }{dt}$	0	0	1
L+F ($O_{1}$ nodal)	145,545	2F + Ω	θ−L−F	$\omega_{E}-\frac{d\left(L+F\right)}{dt}=\omega_{E}-2n+\frac{d\Omega }{dt}$	2	2	1
Semi‐diurnal tides							
$K_{2}$	275,555	Constant	2θ	$2\omega_{E}$	0	0	0
$M_{2}$	255,555	2F+2Ω	2θ−2L	$2\omega_{E}-2n$	2	2	2
$S_{2}$	273,555	2F+2Ω−2D	$2\theta -2L^{\prime }$	$2\omega_{E}-2n^{\prime }$	0	0	0
$N_{2}$	245,655	2F+2Ω+l	2θ−2L−l	$2\omega_{E}-2n-\frac{dl}{dt}=2\omega_{E}-3n+\frac{d\bar{\omega }}{dt}$	3	2	2
Ω ($K_{2}$ nodal)	275,565	Ω	2θ−Ω	$2\omega_{E}-\frac{d\Omega }{dt}$	0	0	1
L+F ($M_{2}$ nodal)	255,545	2F+Ω	2θ−L−F	$2\omega_{E}-\frac{d\left(L+F\right)}{dt}=2\omega_{E}-2n+\frac{d\Omega }{dt}$	2	2	1

^a^ Doodson numbers follow the convention used in J. G. Williams and Boggs (2016, see their page 98) and Petit and Luzum (2010, see their Table 6.7).

Tidal energy dissipation rate depends on the product of the tide‐raising potential and the derivative of the distorted potential. The flow of tidal energy is complicated, but long time averages of the energy dissipation rate are proportional to the squares of the amplitudes of individual tidal constituents. More generally, the tidal energy dissipation rates and tide‐induced Earth‐Moon system evolution rates of Section 5.1 depend on products of linear combinations of the $U_{ij}$ functions times $k_{2mq}\sin\chi_{2mq}$ times angular rates that involve Earth′s rotation rate $\omega_{E}$ and the sidereal mean motion of the Moon n.

The frequencies (ω) of the 14 tidal constituents considered in the orbital dynamics model also are given in Table 1. The sidereal mean motion of the Sun is denoted by $n^{\prime }$. The rate of change of the longitude of perigee, $d\bar{\omega }/dt$, is given by

(4) $$\frac{d\bar{\omega }}{dt}=\frac{3}{4}\frac{{n\prime }^{2}}{n}+\frac{225}{32}\frac{{n\prime }^{3}}{n^{2}}+\frac{4071}{128}\frac{{n\prime }^{4}}{n^{3}}+\frac{265493}{2048}\frac{{n\prime }^{5}}{n^{4}},$$

and the nodal rate dΩ/dt is given by

(5) $$\frac{d\Omega }{dt}=-\frac{3}{4}\frac{{n\prime }^{2}}{n}+\frac{9}{32}\frac{{n\prime }^{3}}{n^{2}}+\frac{273}{128}\frac{{n\prime }^{4}}{n^{3}}.$$

The nodal rate is negative over the parameter space considered in this study.

Three integer sets, $j_{Mq}$, $j_{Wq}$, and $j_{\Omega q}$, are used in some of the expressions in Section 5.1. These integer sets are related to the Delaunay angles in $\zeta_{q}$. In the orbit arguments $\zeta_{q}$, the integer $j_{Mq}$ counts the number of monthly angles, $j_{Wq}$ is associated with dependence on the argument of perigee (L−Ω−l), and $j_{\Omega q}$ is associated with dependence on the node Ω. The values of $j_{Mq}$, $j_{Wq}$, and $j_{\Omega q}$ for the 14 tidal constituents used in the orbital dynamics model are given in Table 1.

## 4 Ocean Tide Model

### 4.1 Description of Ocean Tide Model

The paleotide simulations are run separately from the orbital dynamics model. Due to the much higher computational cost of the ocean tide model, we perform a relatively small number of ocean tide runs, the results of which can then be pulled into the orbital dynamics model runs done later. Specifically, we simulate ocean tides for a discrete set of Earth rotation rates, and for four different basin geometries (PD, 55 Ma, 116 Ma, and 252 Ma), with the model described in Schindelegger et al. (2018). The model accurately computes the self‐attraction and loading (SAL) term (Farrell, 1972; Hendershott, 1972)—described below—inline, as the model steps through time. SAL, a critical component in ocean tide simulations, is often treated iteratively (Arbic, Garner, et al., 2004; Egbert et al., 2004) due to the computational expense of the spherical harmonic transformations underlying an exact SAL solution (Stepanov & Hughes, 2004). Schindelegger et al. (2018) found efficient spherical harmonic solvers, which rendered an inline SAL computation feasible; see also Vinogradova et al. (2015).

For each paleogeometry and discrete Earth rotation rate, ocean tide simulations are separately performed for an $M_{2}$‐like forcing and an $O_{1}$‐like forcing, with the Coriolis parameter and tidal forcing frequency adjusted accordingly. $M_{2}$ is the largest tidal constituent and accounts for about 2/3 of the present‐day tidal energy dissipation rate. $O_{1}$ is the second largest diurnal tidal constituent, and the largest diurnal constituent that is forced solely by the Moon. We make the simplifying assumption that the ocean tide ksinχ factors for the other semi‐diurnal and diurnal tidal constituents included in our orbital dynamics model are equal to those of $M_{2}$ and $O_{1}$, respectively. Because our ocean tide simulations are done with only $M_{2}$ and $O_{1}$, our work here disregards nonlinear interactions between tidal lines of different frequencies, which can be important in coastal areas with strong tidal currents. We also hold the tidal forcing amplitudes (dependent on a) constant, and equal to their present‐day values, in our ocean tide simulations, in order to keep the number of simulations manageable.

The ocean tide simulations employ sidereal Earth rotation rates

(6) $$\omega_{E}=\frac{24\;h}{T}\omega_{E}^{PD},$$

where T is a discrete period from the interval 6–24 hr (in steps of 2 hr) and $\omega_{E}^{PD}$ denotes the present‐day value of $\omega_{E}$ (see Table 4). In the 55 Ma runs, we also perform experiments with T set to 21, 21.5, 22.5, and 23 hr, in order to resolve a resonant $M_{2}$ peak emerging near 22 hr for that particular geometry. In experiments with the present‐day Earth rotation rate, we take the forcing periods of $M_{2}$ and $O_{1}$ to be equal to their present‐day values. In simulations with faster Earth rotation rates, we assume that the $M_{2}$ and $O_{1}$ forcing frequencies are equal to $2\omega_{E}-2n^{PD}$ and $\omega_{E}-2n^{PD}$, respectively, where $n^{PD}$ is the present‐day value of lunar mean motion n, also given in Table 4. The Coriolis parameter is computed from Equation 2. In summary, we use the formulae for the frequencies of $M_{2}$ and $O_{1}$ given in Section 3, but with $\omega_{E}$ adjusted as above and with, for simplicity, n set to its present‐day value in all cases. Later, within the orbital dynamics model, n will take on many values that are different from the present‐day value. However, it is not possible to know ahead of time what these n values will be; hence, we employ the simple assumptions described above.

Schindelegger′s model is based on a one‐layer ocean tide model developed by Einšpigel and Martinec (2017). Here, the model is set up on a 1/8° latitude‐longitude grid ranging from 86°S to 84°N and with vertical walls placed at the northernmost parallel. The ocean tide model solves the shallow‐water equations under the assumptions of incompressibility, constant seawater density, and the Boussinesq approximation (Gill, 1982). Here, for computational efficiency, we turn off the nonlinear momentum advection terms and eddy viscosity terms (see also Green et al., 2017), as is commonly done in barotropic (depth‐averaged) tide models, where the horizontal length scales of interest are large and linear dynamics predominate. The mass conservation equation is:

(7) $$\frac{\partial \zeta }{\partial t}+\nabla \cdot \vec{U}=0,$$

where t is time, ζ is the tidal perturbation sea surface height, and $\vec{U}$ is the depth‐integrated volume transport $\vec{U}=\vec{u}\left(H+\zeta \right)$. Here, $\vec{u}$ represents the two‐dimensional horizontal velocity and H the undisturbed water depth. The momentum equation is:

$$\frac{\partial \vec{U}}{\partial t}+\vec{f}\times \vec{U}=$$

(8) $$-g\left(H+\zeta \right)\nabla \left(\zeta -\zeta_{EQ}-\zeta_{SAL}\right)-{\vec{F}}_{b}-{\vec{F}}_{w},$$

where $\vec{f}=f\hat{k}$ is oriented along the local unit vertical vector $\hat{k}$, and g = 9.80665 m $s^{-2}$ is gravitational acceleration. As is common in ocean models, we neglect the variations in g from equator to pole. The equilibrium tidal forcing $\zeta_{EQ}$, SAL term $\zeta_{SAL}$, quadratic bed friction ${\vec{F}}_{b}$, and spatially constant linear drag ${\vec{F}}_{w}$ are discussed below. To allow for model spin‐up, in all runs performed here, we integrate for 20 days and analyze the last five tidal periods of model output. For each of the four ocean basin geometries used here, initial tests were employed to determine time step sizes required for numerical stability. These time steps are 3.0 s for the 252 Ma geometry, 4.0 s for the 55 Ma geometry, and 4.5 s for the 116 Ma and PD geometries.

The equilibrium tide $\zeta_{EQ}$ (e.g., Cartwright, 1977; Hendershott, 1981) is modified by a factor of $1+k_{2}-h_{2}$. The elastic Love numbers ($h_{2}$ and $k_{2}$, respectively) account for the Earth tide deformation and the perturbation in gravitational potential resulting from this deformation (Farrell, 1972; Hendershott, 1972; Ray, 1998). The Love numbers for the diurnal tides differ from those for the semi‐diurnal and long‐period tides mainly because of the free‐core nutation resonance (Wahr, 1981; Wahr & Sasao, 1981). For a single semi‐diurnal tidal constituent,

(9) $$\zeta_{EQ}=A\;\left[1+k_{2}-h_{2}\right]\;\cos^{2}\phi \;\cos\left[\omega t+2\lambda +V\right],$$

and for a single diurnal constituent,

(10) $$\zeta_{EQ}=A\;\left[1+k_{2}-h_{2}\right]\;\sin\left(2\phi \right)\;\cos\left[\omega t+\lambda +V\right],$$

where ϕ is latitude, and λ is longitude with respect to the Greenwich meridian. If we were to perform a multi‐constituent simulation, V (the phase of the equilibrium tide), A, $k_{2}$, $h_{2}$, and ω, would all take on separate values for each tidal constituent. We ignore the slow nodal modulations of phase and amplitude, which must be considered in more precise analysis and prediction of present‐day tides.

The frequencies, values of A, and values of $1+k_{2}-h_{2}$, for the $M_{2}$ and $O_{1}$ constituents that we directly simulate in this paper, are given in Table 2. For simplicity, all of the ocean tide simulations use present‐day values of A and $1+k_{2}-h_{2}$, taken from the documentation of the TPXO6.2 tidal atlas (Egbert & Erofeeva, 2002). The A values are fixed because it is not feasible to perform simulations for all values of Earth‐Moon distance and other Earth‐Moon system parameters that we will encounter in our integration of the orbital dynamics equations.

**Table 2:** Summary of Astronomical Forcing Parameters and Energetics for Our Ocean Tide Model Simulations^a^ ^,^ ^b^

	$M_{2}$	$O_{1}$
ω	$2\omega_{E}-2n^{PD}$	$\omega_{E}-2n^{PD}$
A (cm)	24.2334	10.0661
$1+k_{2}-h_{2}$	0.693	0.695
KE ($10^{17}$ J)	1.629 (1.779)	0.158 (0.160)
APE ($10^{17}$ J)	1.404 (1.344)	0.127 (0.088)
Energy dissipation rate (TW)	2.514 (2.435)	0.211 (0.173)

^a^ Upper half of the table lists frequencies ω, equilibrium tidal amplitudes A, and Love number combination $1+k_{2}-h_{2}$. $\omega_{E}$ refers to sidereal Earth rotation rate, calculated from present‐day Earth rotation rate $\omega_{E}^{PD}$ (see Table 4) as $\omega_{E}=\left[24\;hours)/T\right]\omega_{E}^{PD}$, where T varies within 6–24 hr. $n^{PD}$ is the present‐day value of lunar mean motion, also given in Table 4.

^b^ Lower half of the table specifies present‐day values of global kinetic energy KE, available potential energy APE, and ocean tide energy dissipation rate, averaged over five tidal cycles in simulations with an optimally tuned constant linear drag. Values in parentheses are the corresponding altimetry‐constrained estimates (Egbert & Ray, 2003) used for comparison and tuning.

The term $\zeta_{SAL}$ combines three effects, the direct gravitational attraction of water toward anomalous water masses (i.e., high or low tides), the deformation of the solid Earth under this anomalous mass loading, and the changes to Earth′s gravitational field resulting from self‐gravitation of the load‐deformed Earth (Farrell, 1972; Hendershott, 1972; Stepanov & Hughes, 2004). These effects are conveniently computed in the spectral domain, by splitting the ocean tide elevations into spherical harmonics, that is,

(11) $$\zeta_{SAL,N}=\sum_{N}\frac{3\rho_{0}}{\rho_{earth}\left(2N+1\right)}\left(1+k_{N}^{\prime }-h_{N}^{\prime }\right)\zeta_{N},$$

where $\rho_{0}$ = 1,035 kg $m^{-3}$ is a mean seawater density, $\rho_{earth}$ = 5,517 kg $m^{-3}$ is the average density of the solid earth, $\zeta_{N}$ refers to the degree N spherical harmonic of the tidal elevation ζ, and $h_{N}^{\prime }$ and $k_{N}^{\prime }$ are the degree‐dependent load Love numbers, introduced by W. H. Munk and MacDonald (1960). As in Schindelegger et al. (2018), we use load Love numbers from Wang et al. (2012) and transform degrees N={0,1} to the center‐of‐figure frame. For Equation 11 to be weaved into the time‐stepping procedure, the model must be capable of expanding instantaneous tidal elevations ζ into spherical harmonics, evaluating Equation 11, and transforming the so‐derived spherical harmonic representation of SAL ($\zeta_{SAL,N}$) back to the model grid. We employ a high‐performance spherical harmonic library (Schaeffer, 2013) to accomplish these tasks at each time step. The decomposition into $\zeta_{N}$ is truncated at degree N=719, equivalent to a spatial resolution of 1/4°.

Damping terms are another important component of tide models (see Arbic, Garner, et al., 2004; Egbert et al., 2004). The quadratic bed friction term (Taylor, 1919) ${\vec{F}}_{b}=C_{d}\vec{U}|\vec{u}|/\left(H+\zeta \right)$, with a dimensionless drag coefficient $C_{d}$ set to 0.0025, is standard in the tidal modeling literature, and predominantly dissipates energy on shelves characterized by strong tidal flows (e.g., Egbert & Ray, 2000; Jayne & St Laurent, 2001). Since the work of Jayne and St Laurent (2001), most barotropic tide models also have employed a parameterized topographic wave drag, which accounts for the breaking of internal tides that are generated by barotropic tidal flow over topographic features such as abyssal hills, seamounts, and shelf breaks (Egbert & Ray, 2000, 2001, 2003). Internal tides, a subject of intense interest in modern physical oceanography, are undulations of tidal frequency that lie on the interfaces of fluids of different density. See Arbic et al. (2018) and references therein for a review of global internal tide modeling.

There are many complexities involved in parameterizing wave drag for paleotide models. In most studies in the literature, wave drag acts linearly on tidal velocity, but with a strength that varies spatially according to stratification and topographic roughness. The spatial patterns of diurnal and semi‐diurnal tidal energy dissipation rate differ from each other (Egbert & Ray, 2003; Skiba et al., 2013). Therefore, a wave drag scheme tuned for the $M_{2}$ constituent is not strictly appropriate for $O_{1}$. Furthermore, any wave drag parameterization we might choose to employ would not produce significant drag in the paleogeometries that are overly smooth. In the face of these difficulties, we employ a spatially constant linear drag ${\vec{F}}_{w}=C_{w}\vec{U}$ as a stand‐in for parameterized topographic wave drag. We prescribe the same strength coefficient $C_{w}$ ($s^{-1}$) in simulations of $M_{2}$ and $O_{1}$. The use of a spatially constant linear drag bypasses the complexities of changing bathymetric roughnesses and bottom stratifications throughout deep time. Green et al. (2017) found only small sensitivities of deep‐time tide models to the changes in drag strength that would result from changes in stratification. The assumption of relatively constant seafloor roughness throughout deep‐time is more problematic, but is made here for simplicity. The $C_{w}$ value was determined from tuning experiments under present‐day conditions, with estimates of elevations, energies, and energy dissipation rates from satellite altimetry (Egbert & Erofeeva, 2002; Egbert & Ray, 2003) adopted as benchmarks. Simulations with $C_{w}$ values of order (2.5 days)^−1^ produce globally integrated $M_{2}$ and $O_{1}$ energies closest to the observations. Estimates of the global energy dissipation rate for each constituent are rather insensitive to the choice of $C_{w}$, as a higher weight on the linear drag typically is compensated by less dissipation due to quadratic bed friction.

The globally integrated tidal energy dissipation rates are computed during each simulation (at 15‐min intervals) as the sum of the dissipation rates from ${\vec{F}}_{b}$ and ${\vec{F}}_{w}$. We average the resulting time series over the last five forcing cycles of the respective constituent and compare this estimate of total tidal energy dissipation rate to the tidal power input $P_{in}$, given by

(12) $$P_{in}=\rho_{0}g\int \;\;\int <\zeta_{EQ}\frac{\partial \zeta }{\partial t}>\;dA$$

(Egbert & Ray, 2001, and references therein), where dA is an element of area, and angle brackets indicate time‐averaging. In all of the ocean tide simulations performed for this study, the power inputs and energy dissipation rates agree to within 1.7%, and the SAL term contributes less than 0.6% to the energy budget. This level of agreement between power input and energy dissipation rate, and the small contributions of the SAL term to the energy budget, are more than satisfactory for the present study.

The global time‐averaged energy dissipation rates, kinetic energies (KE), and available potential energies (APE), from our preferred present‐day $M_{2}$ and $O_{1}$ simulations with $C_{w}$ set to (2.5 days)^−1^, are given in Table 2. The modeled present‐day $O_{1}$ dissipation rate is within ∼20% of the rate inferred from satellite altimetry, and the modeled present‐day $M_{2}$ rate is much closer to altimeter values. Instantaneous values of globally integrated KE and APE are computed as

(13) $$KE=\frac{1}{2}\rho_{0}\int \;\;\int \left(H+\zeta \right)\vec{u}\cdot \vec{u}\;dA,\;APE=\frac{1}{2}\rho_{0}g\int \;\;\int \zeta^{2}\;dA,$$

and time‐averaging is then applied to obtain the values in Table 2. The modeled present‐day $O_{1}\;APE$ value differs from the satellite altimeter value by 44%, while the other three model KE and APE values given in Table 2 differ from satellite altimeter values by 9% or less.

In order to display spatial maps of the tidal amplitudes in selected runs, we employ standard tidal harmonic analysis. This allows us to write the tidal elevations ζ at each grid point in terms of amplitude and phase lags, viz.

(14) ζ(ϕ,λ)=Amplitude(ϕ,λ)cos[ωt−Phase(ϕ,λ)].

### 4.2 Ocean Tide Model Results

**Figure 2:**
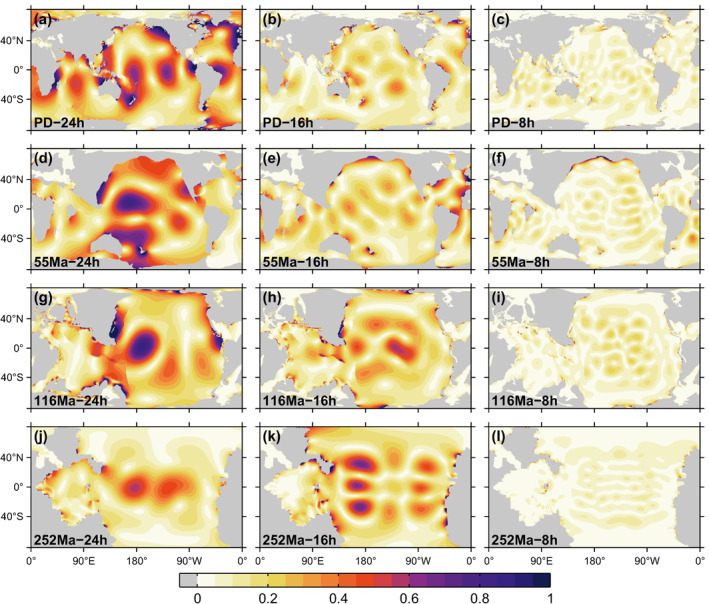
Global maps of $M_{2}$ amplitude (m) with present‐day (PD), 55 Ma, 116 Ma, and 252 Ma bathymetries (top to bottom rows, respectively), and T values (in Equation 6) of 24, 16, and 8 hr (left, middle, and right columns, respectively).

Maps of $M_{2}$ tidal amplitudes, for T values (Equation 6) of 24, 16, and 8 hr, astronomical forcing amplitudes $A\left(1+k_{2}-h_{2}\right)$ equal to present‐day values, and the four basin geometries, are given in Figure 2. For T = 24 hr, there is a substantial decrease in North Atlantic amplitudes from the PD to 55 Ma geometry, consistent with results from Green and Huber (2013). Evidently, because the 55 Ma Atlantic is narrower, the Atlantic ocean is farther from resonance for the $M_{2}$ tide. The large Pacific tides in the 55 Ma, T = 24 hr simulation are insufficient to keep the 55 Ma energy dissipation rate at the present‐day level. For all four geometries, $M_{2}$ tidal amplitudes generally decrease as the rotation periods decrease. The 252 Ma, T = 16 hr simulation represents an exception, with larger amplitudes than are seen in the 252 Ma, T = 24 hr simulation. Visual inspection of Figure 2 indicates decreased horizontal length scales as the value of T decreases, consistent with the expected decrease in Rossby deformation radius ($\sqrt{gH}/f$) as f increases with increasing Earth rotation rates. Maps of the $O_{1}$ amplitudes (not shown for the sake of brevity) also display noticeably shorter horizontal length scales and smaller amplitudes as rotation rates increase.

**Figure 3:**
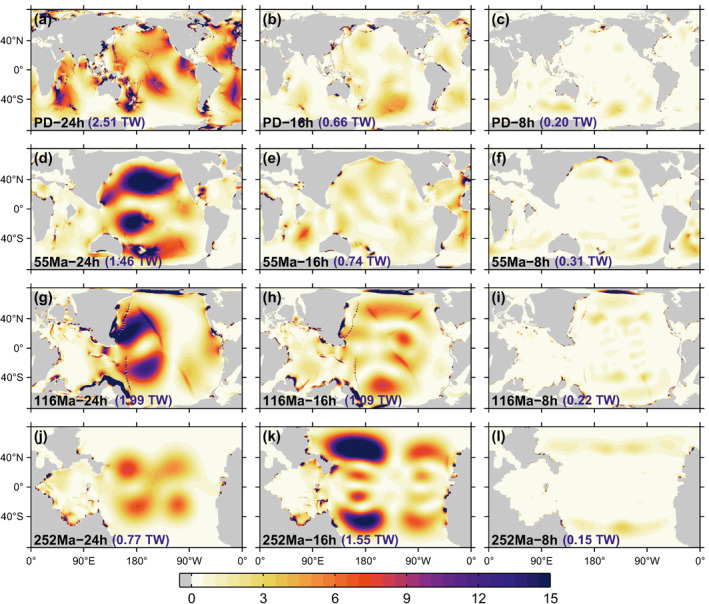
As in Figure 2, but for $M_{2}$ energy dissipation rate maps (mW $m^{-2}$). Numbers given in parentheses at the bottom of each subplot present the globally integrated $M_{2}$ ocean tidal dissipation rate (TW) of each simulation. Results for T = 24 hr (leftmost panels) can be compared with Green et al. (2017), who found total ocean tide dissipation rates of $\left(1.44,2.12,0.90\right)$ TW for the three paleo geometries, using another ocean model with a different treatment of self‐attraction and loading and wave drag.

Corresponding $M_{2}$ energy dissipation rate maps are given in Figure 3. The smaller tidal amplitudes generally seen in Figure 2 with increasing Earth rotation rate translate into smaller energy dissipation rates in Figure 3. Once again, the 252 Ma, T = 16 hr simulation, which has larger dissipation rates than the 252 Ma, T = 24 hr simulation, represents a notable exception. Inspection of the energy dissipation rate maps in the T = 24 hr simulations suggests that regions of large dissipation rate over present‐day continental shelf areas are not as prominent in the paleogeometries. We speculate that this may be due to the smoother shorelines and relative lack of indented bays in the paleogeometries, the latter associated in part with the lack of glaciation during those epochs. The $O_{1}$ dissipation maps are not shown but display similar trends as the $M_{2}$ maps.

**Figure 4:**
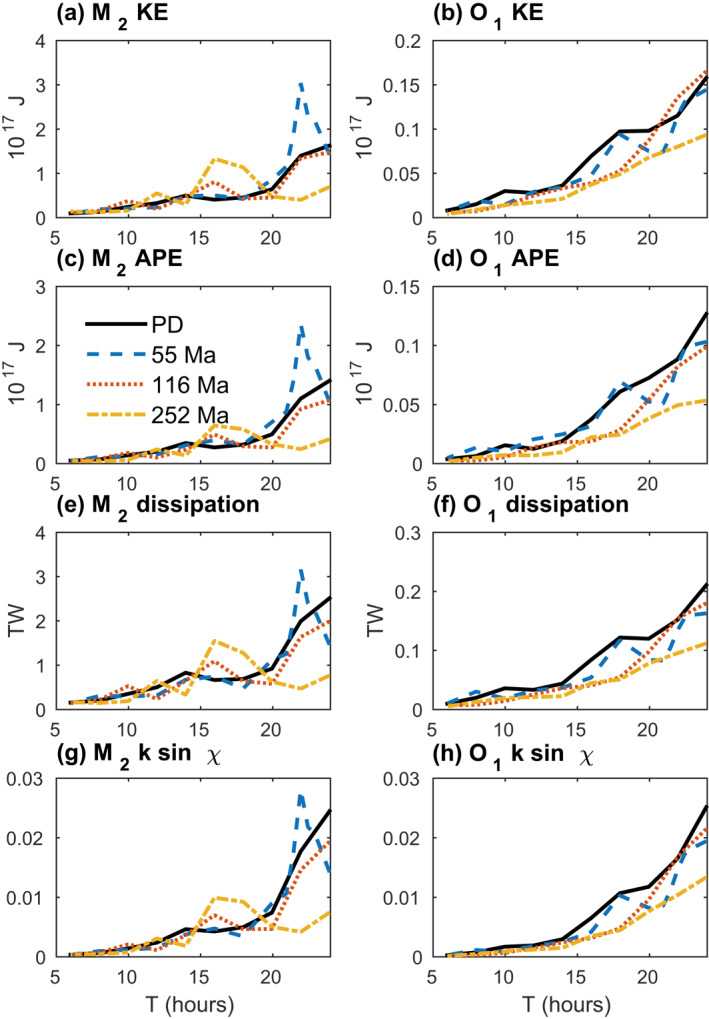
Globally integrated kinetic energy (KE; a and b), available potential energy (APE; c and d), energy dissipation rate (e and f), and ksinχ values (g and h), for $M_{2}$ (left‐hand side subplots) and $O_{1}$ (right‐hand side subplots) ocean tide simulations with different values of T in Equation 6, and the four bathymetries shown in Figure 1. Four additional T values were used in the 55 Ma simulations, in order to better resolve the peak near 22 hr. Globally integrated KE and APE values are computed via Equation 13. This figure, and all subsequent figures, have legends, and the legends are not enclosed by boxes.

The global $M_{2}$ and $O_{1}\;KE$, APE, and energy dissipation rates, and the ksinχ values inferred from the global energy dissipation rates, display a general tendency to become smaller as Earth′s rotation rate increases (Figure 4). The energies, energy dissipation rates, and ksinχ values are computed from simulations performed with all four basin geometries, and with the discrete set of T values from 6–24 hr. The $M_{2}$ and $K_{1}\;k\sin\chi$ values are computed from the dissipation rates via Equations 20 and 22, respectively, assuming a present‐day value of a and the appropriate value of $\omega_{E}$ (Table 2). The $M_{2}$ PD simulations yield especially significant drops in dissipation rate and ksinχ values as T is reduced from 24 to 22 to 20 hr. Again, an exception to these general tendencies is seen in the 252 Ma simulation with T = 16 hr. Another departure from the trend toward smaller values with decreasing T values is seen in the 55 Ma T = 22 hr simulation. The observation of reduced tides and tidal dissipation rates with increased Earth rotation rate is in qualitative agreement with the results of Webb (1982). As a reminder, in our ocean tide simulations, the astronomical forcing amplitudes (which depend on a) are kept at present‐day values. Hence, a decrease in ksinχ values in the simulations implies a decrease in tidal energy dissipation rates. However, as noted earlier, reduced ksinχ values do not necessarily imply a reduced energy dissipation rate in true deep‐time conditions, for which the semi‐major axis a of the lunar orbit can decrease substantially. The reduction of a can potentially overcome the decrease in ksinχ, such that the ocean dissipation rate may increase even if the proportionality factor ksinχ decreases. We will return to this point later in the study.

## 5 Description of Orbital Dynamics Model

The equations for tidal evolution of the lunar orbit, Earth rotation rate and obliquity, and lunar orientation are coupled. This section presents a set of differential equations, taken from J. G. Williams and Boggs (2016) and J. G. Williams et al. (2001), that govern this evolution. The differential equations are based on a precessing elliptical lunar orbit. For the perturbed orbit, the equations presented here should be accurate to within a few percent. The equations account for secular changes in Earth′s rotation rate $\omega_{E}$, the semi‐major axis a of the geocentric lunar orbit, Earth′s obliquity ε (the angle made by Earth′s equator plane to the ecliptic plane), the inclination i of the Moon′s orbital plane to the ecliptic plane, and the eccentricity e of the lunar orbit. The secular changes of node Ω and longitude of perigee $\bar{\omega }$ were given in Section 3. The equations account for the effects of tides raised on Earth (in both the solid Earth and ocean) by the Moon and Sun, tides raised within the Moon by Earth, and core‐mantle boundary (CMB) dissipation in the Moon.

The equations governing secular changes in the state of the Earth‐Moon system do not model the instantaneous evolution. Accurate equations for instantaneous positions must account for the influence of the Sun′s gravity, are more complicated than the secular equations (Park et al., 2021), and are critical to the success of modern laser ranging measurements of cm/year‐scale secular changes in the semi‐major axis a. Although the non‐secular deviations are large (of order several thousand kilometers), they are not expected to greatly affect the secular changes that we focus on here.

### 5.1 Evolution due to Tides on Earth

Each phase shifted component of the tidal bulge will apply a torque between the Moon and Earth. Because Earth spins faster than the Moon′s mean motion ($\omega_{E}>n$), the bulge from diurnal and semi‐diurnal tides leads the lunar position and the torque retards Earth′s rotation rate and accelerates the Moon forward. The bulge from long period (zonal) tides is not shifted by Earth′s rotation. The secular change in Earth′s rotation rate due to diurnal and semi‐diurnal tides on Earth is given by

(15) $$\begin{array}{ccc} \frac{d\omega_{E}}{dt}=-\frac{3}{8}\frac{GM_{M}^{2}}{M_{E}a^{3}}{\left(\frac{R}{a}\right)}^{3}\frac{M_{E}R^{2}}{S_{W}C\left(\omega_{E}\right)}\sum_{q}\left(1+\delta_{q0}\right) & & \end{array}$$

$$\begin{array}{ccc} \times \left\{\begin{array}{c} 2{\left[U_{13q}-U_{23q}\right]}^{2}k_{21q-}\sin\chi_{21q-}\;\\ +2{\left[U_{13q}+U_{23q}\right]}^{2}k_{21q+}\sin\chi_{21q+}\;\\ +{\left[U_{11q}-U_{22q}+2U_{12q}\right]}^{2}k_{22q-}\sin\chi_{22q-}\;\\ +{\left[U_{11q}-U_{22q}-2U_{12q}\right]}^{2}k_{22q+}\sin\chi_{22q+},\; \end{array}\right. & & \end{array}$$

where G is the gravitational constant, $M_{M}$ and $M_{E}$ respectively denote the masses of the Moon and Earth, R is Earth′s equatorial radius, and $C\left(\omega_{E}\right)$ is Earth′s polar principal moment of inertia, given by

(16) $$C\left(\omega_{E}\right)=\left[1+0.655\times 10^{6}\omega_{E}^{2}\left(\frac{2k_{2f}}{3}+s_{f}\right)\right]C\left(0\right),$$

where in this equation $\omega_{E}$ must be given in radians/second. The parameters $k_{2f}$ and $s_{f}$ are related to the dependence of the moment of inertia about the polar axis on both spherical and oblate distortions. The fluid Love number $k_{2f}$ describes the oblate distortion of degree two, and $s_{f}$ describes the degree‐0 spherical distortion (Dahlen, 1976; J. G. Williams et al., 2001). The moment for zero spin rate is denoted by C(0). The value of C(0) for the present‐day Earth, with an equatorial mean radius of 6,371 km, is 0.3299$M_{E}R^{2}$. A homogeneous sphere would have C(0) = 0.4$M_{E}R^{2}$. The parameter

(17) $$S_{W}=\frac{\frac{d}{dt}\left[C\left(\omega_{E}\right)\omega_{E}\right]}{C\left(\omega_{E}\right)\frac{d\omega_{E}}{dt}}=1+\frac{\omega_{E}\frac{d}{dt}\left[C\left(\omega_{E}\right)\right]}{C\left(\omega_{E}\right)\frac{d\omega_{E}}{dt}}=1+1.311\times 10^{6}\omega_{E}^{2}\left(\frac{2k_{2f}}{3}+s_{f}\right)$$

allows for the small dependence of the moment of inertia (Equation 16) on Earth's rotation rate.

Diurnal tides (degree 2, order 1) have subscripts (_21_) and semi‐diurnal tides have subscripts (_22_). The Kronecker delta $\delta_{q0}$ is 1 when q = 0 and is 0 when q≠ 0. The $1+\delta_{q0}$ factor in Equation 15 arises because squaring sines or cosines or multiplying sines and cosines together introduces a factor of 1/2. The $K_{1}$, $K_{2}$, and long period tides do not have eastward and westward components, which reduces the number of trigonometric multiplications and the factors of 1/2. Equation 15 is written with eastward and westward propagating tides. The $K_{1}$ and $K_{2}$ tides with q=0 are split into two parts with “+” and “−” subscripts, but they have the same phase and amplitude and are two halves of the same tides. The q≠0 eastward propagating tides are small and are ignored in this study. Consequently, if the tides with subscript “+” are removed from Equation 15, then the $\left(1+\delta_{q0}\right)$ factor for $K_{1}$ and $K_{2}$ should be squared. Long‐period tidal constituents (with subscript 20q) do not produce secular changes in Earth's rotation rate $\omega_{E}$. The $U_{13q}-U_{23q}$ Cartesian factors for the diurnal tidal constituents used in our orbital dynamics model are given by

(18) $$\left\{\begin{array}{c} K_{1}\;\mathrm{lunar}\;\mathrm{part}:-\frac{1}{2}\sin\varepsilon \cos\varepsilon \frac{\left[1-\frac{3}{2}\sin^{2}i\right]}{{\left(1-e^{2}\right)}^{1.5}},\;\\ K_{1}\;\mathrm{solar}\;\mathrm{part}:-\frac{1}{2}\frac{M_{S}a^{3}}{M_{M}{a\prime }^{3}}\frac{\sin\varepsilon \cos\varepsilon }{{\left(1-{e\prime }^{2}\right)}^{1.5}},\;\\ O_{1}:\frac{{\left(1+\cos i\right)}^{2}}{8}\sin\varepsilon (1+\cos\varepsilon )\left[1-\frac{5e^{2}}{2}+\frac{13e^{4}}{16}\ldots \right],\;\\ P_{1}:\frac{1}{2}\frac{M_{S}a^{3}}{M_{M}{a\prime }^{3}}\sin\varepsilon (1+\cos\varepsilon )\left[1-\frac{5{e\prime }^{2}}{2}+\frac{13{e\prime }^{4}}{16}\ldots \right],\;\\ Q_{1}:\frac{{\left(1+\cos i\right)}^{2}}{8}\sin\varepsilon (1+\cos\varepsilon )\left[\frac{7e}{2}-\frac{123e^{3}}{16}\ldots \right],\;\\ \Omega \;(K_{1}\;\mathrm{nodal}):-\frac{1}{2}\sin i\cos i(2\cos\varepsilon -1)(1+\cos\varepsilon )\frac{1}{{\left(1-e^{2}\right)}^{1.5}},\;\\ L+F\;(O_{1}\;\mathrm{nodal}):\frac{1}{4}\sin i(1+\cos i)(2\cos\varepsilon -1)(1+\cos\varepsilon )\left[1-\frac{5e^{2}}{2}+\frac{13e^{4}}{16}\ldots \right], \end{array}\right.$$

while the $U_{11q}-U_{22q}+2U_{12q}$ Cartesian factors for the semi‐diurnal tidal constituents that we employ are

(19) $$\left\{\begin{array}{c} K_{2}\;\mathrm{lunar}\;\mathrm{part}:\frac{1}{2}\sin^{2}\varepsilon \frac{\left[1-\frac{3}{2}\sin^{2}i\right]}{{\left(1-e^{2}\right)}^{1.5}},\;\\ K_{2}\;\mathrm{solar}\;\mathrm{part}:\frac{1}{2}\frac{M_{S}a^{3}}{M_{M}{a\prime }^{3}}\frac{\sin^{2}\varepsilon }{{\left(1-{e\prime }^{2}\right)}^{1.5}},\;\\ M_{2}:\frac{{\left(1+\cos i\right)}^{2}}{8}{\left(1+\cos\varepsilon \right)}^{2}\left[1-\frac{5e^{2}}{2}+\frac{13e^{4}}{16}\ldots \right],\;\\ S_{2}:\frac{1}{2}\frac{M_{S}a^{3}}{M_{M}{a\prime }^{3}}{\left(1+\cos\varepsilon \right)}^{2}\left[1-\frac{5{e\prime }^{2}}{2}+\frac{13{e\prime }^{4}}{16}\ldots \right],\;\\ N_{2}:\frac{{\left(1+\cos i\right)}^{2}}{8}{\left(1+\cos\varepsilon \right)}^{2}\left[\frac{7e}{2}-\frac{123e^{3}}{16}\ldots \right],\;\\ \Omega \;(K_{2}\;\mathrm{nodal}):\sin i\cos i\sin\varepsilon (1+\cos\varepsilon )\frac{1}{{\left(1-e^{2}\right)}^{1.5}},\;\\ L+F\;(M_{2}\;\mathrm{nodal}):-\frac{1}{2}\sin i(1+\cos i)\sin\varepsilon (1+\cos\varepsilon )\left[1-\frac{5e^{2}}{2}+\frac{13e^{4}}{16}\ldots \right]. \end{array}\right.$$

The Cartesian terms for solar tidal constituents such as $P_{1}$ and $S_{2}$, and the terms for the solar parts of $K_{1}$ and $K_{2}$, include a factor of $\left[M_{S}a^{3}]/[M_{M}{a\prime }^{3}\right]$, where $M_{S}$ is the mass of the Sun and $a^{\prime }$ is the semi‐major axis of Earth′s orbit around the Sun. The semi‐major axis $a^{\prime }$ of Earth′s orbit changes little in time and is taken to be constant in this study. (The constancy of $a^{\prime }$ in our model implies that the period of Earth′s orbit around the Sun is constant. However, because Earth′s rotation rate is changing, the number of days in a year was greater in the distant past). In the case of tidal constituents $K_{1}$ and $K_{2}$, the Cartesian factors of the solar and lunar components are added together before the expression is used in our equations. The eccentricity of Earth′s orbit around the Sun, $e^{\prime }$, enters into the expressions in Equations 18 and 19. The functions of eccentricity are the G functions in Kaula (1966, Chapter 3). The variable $e^{\prime }$ does change significantly over time scales of order hundreds of Ma (Laskar et al., 2004). In this study, we take $e^{\prime }$ = 0.0316, the root mean square value of $e^{\prime }\left(t\right)$ over long time spans.

The ocean tide modeling literature focuses on tidal power dissipation, whereas the orbital dynamics literature tends to discuss tidal friction in terms of the potential Love number $k_{2}$, and frictional phase lags χ (or, alternatively, the quality factor Q). Equations relating tidal dissipation to the Love number factors $k_{22q-}\sin\chi_{22q-}$ for westward propagating semi‐diurnal tides, $k_{21q-}\sin\chi_{21q-}$ for westward propagating diurnal tides, and $k_{20q}\sin\chi_{20q}$ for long‐period tides, are given below. For all tidal constituents, the ksinχ values contain both ocean and Earth tide contributions. For semi‐diurnal tides, the relationship between tidal energy dissipation rate and the ksinχ factor is:

(20) $$\begin{array}{ccc} {Dissipation}_{22}=\frac{3}{16}\frac{GM_{M}^{2}}{a}{\left(\frac{R}{a}\right)}^{5}\sum_{q}\left[2\omega_{E}{\left(1+\delta_{q0}\right)}^{2}-j_{Mq}nS_{A}\right] & & \\ \times {\left[U_{11q}-U_{22q}+2U_{12q}\right]}^{2}k_{22q-}\sin\chi_{22q-}, & & \end{array}$$

where, once again, the smaller eastward propagating semi‐diurnal tides have been ignored. The term $S_{A}$, a small refinement over the Keplerian case, is defined by

(21) $$S_{A}=1+\frac{2{n\prime }^{2}}{n^{2}}.$$

The relationship for diurnal tides is

(22) $$\begin{array}{ccc} {Dissipation}_{21}=\frac{3}{4}\frac{GM_{M}^{2}}{a}{\left(\frac{R}{a}\right)}^{5}\sum_{q}\left[\omega_{E}{\left(1+\delta_{q0}\right)}^{2}-j_{Mq}nS_{A}\right]{\left[U_{13q}-U_{23q}\right]}^{2} & & \\ \times k_{21q-}\sin\chi_{21q-}, & & \end{array}$$

and for long‐period tides is

(23) $${Dissipation}_{20}=nS_{A}\frac{GM_{M}^{2}}{8a}{\left(\frac{R}{a}\right)}^{5}\sum_{q}j_{Mq}{\left[U_{11q}+U_{22q}-2U_{33q}\right]}^{2}k_{20q}\sin\chi_{20q},$$

where the $U_{11q}+U_{22q}-2U_{33q}$ terms for the two largest long period constituents are given by

(24) $$\left\{\begin{array}{c} M_{f}:\frac{3{\left(1+\cos i\right)}^{2}}{8}\sin^{2}\varepsilon \left[1-\frac{5e^{2}}{2}+\frac{13e^{4}}{16}\ldots \right],\;\\ M_{m}:2\left(1-\frac{3}{2}\sin^{2}i\right)\left(1-\frac{3}{2}\sin^{2}\varepsilon \right)\left[\frac{3e}{2}+\frac{27e^{3}}{16}\ldots \right].\; \end{array}\right.$$

The energy dissipation rate formulae for all three species of tides—semi‐diurnal, diurnal, and long‐period—contain terms that are proportional to the lunar mean motion n. The dissipation formulae for the semi‐diurnal and diurnal tides also contain terms that are proportional to Earth′s rotation rate, $\omega_{E}$. These results highlight the fact that the dissipated energy is the energy drawn from Earth rotation minus energy going into the lunar orbit. The semi‐diurnal tidal energy dissipation rate is proportional to the squares of the Cartesian factors $U_{11q}-U_{22q}+2U_{12q}$ for the different tidal constituents. Similarly, the diurnal tidal energy dissipation rate is proportional to the squares of the Cartesian factors $U_{13q}-U_{23q}$, and the long‐period tidal energy dissipation rate is proportional to the squares of the Cartesian factors $U_{11q}+U_{22q}-2U_{33q}$. The semi‐diurnal and diurnal energy dissipation rates contain factors of ${\left(1+\delta_{q0}\right)}^{2}$ in the terms associated with the Earth rotation rate $\omega_{E}$. This is because, as discussed above, we are only considering westward propagating tides in Equations 20 and 22.

Following J. G. Williams and Boggs (2016), the change in Earth's obliquity, dε/dt, due to tides on Earth, is divided into three components: $d\varepsilon_{20}/dt$ (obliquity changes due to long‐period tides), $d\varepsilon_{21}/dt$ (obliquity changes due to diurnal tides), and $d\varepsilon_{22}/dt$ (obliquity changes due to semi‐diurnal tides), viz.

(25) $$\frac{d\varepsilon_{20}}{dt}=-\frac{3}{4}\frac{GM_{M}^{2}}{M_{E}a^{3}}{\left(\frac{R}{a}\right)}^{3}\frac{M_{E}R^{2}}{C\left(\omega_{E}\right)\omega_{E}}\sum_{q}\left(1+\delta_{q0}\right)U_{13q}\left[U_{11q}+U_{22q}-2U_{33q}\right]k_{20q}\sin\chi_{20q},$$

(26) $$\begin{array}{c} \frac{d\varepsilon_{21}}{dt}=\frac{3}{4}\frac{GM_{M}^{2}}{M_{E}a^{3}}{\left(\frac{R}{a}\right)}^{3}\frac{M_{E}R^{2}}{C\left(\omega_{E}\right)\omega_{E}}\sum_{q}\left(1+\delta_{q0}\right) \end{array}$$

$$\times \left\{\begin{array}{c} \left(U_{11q}-U_{33q}+U_{12q}\right)\left(U_{13q}-U_{23q}\right)k_{21q-}\sin\chi_{21q-}\;\\ -\left(U_{11q}-U_{33q}-U_{12q}\right)\left(U_{13q}+U_{23q}\right)k_{21q+}\sin\chi_{21q+}, \end{array}\right.$$

(27) $$\begin{array}{ccc} \frac{d\varepsilon_{22}}{dt}=\frac{3}{8}\frac{GM_{M}^{2}}{M_{E}a^{3}}{\left(\frac{R}{a}\right)}^{3}\frac{M_{E}R^{2}}{C\left(\omega_{E}\right)\omega_{E}}\sum_{q}\left(1+\delta_{q0}\right) & & \end{array}$$

$$\begin{array}{c} \times \left\{\begin{array}{c} \left(U_{13q}-U_{23q}\right)\left(U_{11q}-U_{22q}+2U_{12q}\right)k_{22q-}\sin\chi_{22q-}\;\\ -\left(U_{13q}+U_{23q}\right)\left(U_{11q}-U_{22q}-2U_{12q}\right)k_{22q+}\sin\chi_{22q+}.\; \end{array}\right. \end{array}$$

Additional Cartesian factors $U_{13q}$ are needed for secular obliquity changes from long period tides. The $U_{13}$ Cartesian factors for $M_{f}$ and $M_{m}$, the two largest long‐period tides, are

(28) $$\left\{\begin{array}{c} M_{f}:\frac{{\left(1+\cos i\right)}^{2}}{8}\sin\varepsilon \left[1-\frac{5e^{2}}{2}+\frac{13e^{4}}{16}\ldots \right],\;\\ M_{m}:0.\; \end{array}\right.$$

The $U_{13}$ factor for the zero‐frequency long‐period tide is 0. Cartesian factors $U_{11q}-U_{33q}+U_{12q}$ are needed for secular obliquity changes from diurnal tides. The values of these Cartesian factors for the diurnal tides employed in our orbital model are

(29) $$\left\{\begin{array}{c} K_{1}\;\mathrm{lunar part}:\frac{1}{2}\cos^{2}\varepsilon \left(1-\frac{3}{2}\sin^{2}i\right)\frac{1}{{\left(1-e^{2}\right)}^{1.5}},\;\\ K_{1}\;\mathrm{solar part}:\frac{1}{2}\frac{M_{S}a^{3}}{M_{M}{a\prime }^{3}}\cos^{2}\varepsilon \frac{1}{{\left(1-{e\prime }^{2}\right)}^{1.5}},\;\\ O_{1}:\frac{1}{8}(2-\cos\varepsilon )(1+\cos\varepsilon ){\left(1+\cos i\right)}^{2}\left[1-\frac{5e^{2}}{2}+\frac{13e^{4}}{16}\ldots \right],\;\\ P_{1}:\frac{1}{2}\frac{M_{S}a^{3}}{M_{M}{a\prime }^{3}}(2-\cos\varepsilon )(1+\cos\varepsilon )\left[1-\frac{5{e\prime }^{2}}{2}+\frac{13{e\prime }^{4}}{16}\ldots \right],\;\\ Q_{1}:\frac{{\left(1+\cos i\right)}^{2}}{8}(2-\cos\varepsilon )(1+\cos\varepsilon )\left[\frac{7e}{2}-\frac{123e^{3}}{16}\ldots \right],\;\\ \Omega \;(K_{1}\;\mathrm{nodal}):-\frac{1}{2}\sin i\cos i\sin\varepsilon (2\cos\varepsilon -1)\frac{1}{{\left(1-e^{2}\right)}^{1.5}},\;\\ L+F\;(O_{1}\;\mathrm{nodal}):\frac{1}{4}\sin i(1+\cos i)\sin\varepsilon (2\cos\varepsilon -1)\left[1-\frac{5e^{2}}{2}+\frac{13e^{4}}{16}\ldots \right].\; \end{array}\right.$$

The $U_{11q}-U_{33q}+U_{12q}$ Cartesian factors for $P_{1}$ and for the solar part of $K_{1}$ are adjusted by $\left[M_{S}a^{3}]/[M_{M}{a\prime }^{3}\right]$. Cartesian factors $U_{13q}-U_{23q}$ are needed for secular obliquity changes from semi‐diurnal tides. The $U_{13q}-U_{23q}$ factors for the semi‐diurnal tides employed in our orbital model are

(30) $$\left\{\begin{array}{c} K_{2}\;\mathrm{lunar}\;\mathrm{part}:-\frac{1}{2}\sin\varepsilon \cos\varepsilon \frac{\left[1-\frac{3}{2}\sin^{2}i\right]}{{\left(1-e^{2}\right)}^{1.5}},\;\\ K_{2}\;\mathrm{solar}\;\mathrm{part}:-\frac{1}{2}\frac{M_{S}a^{3}}{M_{M}{a\prime }^{3}}\frac{\sin\varepsilon \cos\varepsilon }{{\left(1-{e\prime }^{2}\right)}^{1.5}},\;\\ M_{2}:\frac{{\left(1+\cos i\right)}^{2}}{8}\sin\varepsilon (1+\cos\varepsilon )\left[1-\frac{5e^{2}}{2}+\frac{13e^{4}}{16}\ldots \right],\;\\ S_{2}:\frac{1}{2}\frac{M_{S}a^{3}}{M_{M}{a\prime }^{3}}\sin\varepsilon (1+\cos\varepsilon )\left[1-\frac{5{e\prime }^{2}}{2}+\frac{13{e\prime }^{4}}{16}\ldots \right],\;\\ N_{2}:\frac{{\left(1+\cos i\right)}^{2}}{8}\sin\varepsilon (1+\cos\varepsilon )\left[\frac{7e}{2}-\frac{123e^{3}}{16}\ldots \right],\;\\ \Omega \;(K_{2}\;\mathrm{nodal}):-\frac{1}{2}\sin i\cos i(2\cos\varepsilon -1)(1+\cos\varepsilon )\frac{1}{{\left(1-e^{2}\right)}^{1.5}},\;\\ L+F\;(M_{2}\;\mathrm{nodal}):\frac{1}{4}\sin i(1+\cos i)(2\cos\varepsilon -1)(1+\cos\varepsilon )\left[1-\frac{5e^{2}}{2}+\frac{13e^{4}}{16}\ldots \right],\; \end{array}\right.$$

which correspond to the $U_{13q}-U_{23q}$ factors for the corresponding diurnal tides, given in Equation 18. Again, in practice we ignore eastward propagating terms in equations for dε/dt, and therefore a squared factor of $\left(1+\delta_{q0}\right)$ is used for $K_{1}$ and $K_{2}$, in place of the pair of eastward and westward motions. We also will ignore eastward propagating terms in equations for the rates of change da/dt, de/dt, and di/dt due to tides on Earth.

The equation for the change in semi‐major axis, da/dt due to tides on Earth, is

(31) $$\begin{array}{ccc} \frac{da}{dt}=\frac{naS_{A}}{2}\frac{M_{M}}{M_{E}}{\left(\frac{R}{a}\right)}^{5}\sum_{q}j_{Mq} & & \end{array}$$

$$\begin{array}{ccc} \times \left\{\begin{array}{c} -\frac{1}{2}{\left[U_{11q}+U_{22q}-2U_{33q}\right]}^{2}k_{20q}\sin\chi_{20q}\;\\ +3{\left[U_{23q}-U_{13q}\right]}^{2}k_{21q-}\sin\chi_{21q-}\;\\ -3{\left[U_{23q}+U_{13q}\right]}^{2}k_{21q+}\sin\chi_{21q+}\;\\ +\frac{3}{4}{\left[U_{11q}-U_{22q}+2U_{12q}\right]}^{2}k_{22q-}\sin\chi_{22q-}\;\\ -\frac{3}{4}{\left[U_{11q}-U_{22q}-2U_{12q}\right]}^{2}k_{22q+}\sin\chi_{22q+},\; \end{array}\right. & & \end{array}$$

while the equations for de/dt and di/dt due to tides on Earth are, respectively,

(32) $$\begin{array}{ccc} \frac{de}{dt}=\frac{nS_{E}}{4e}\frac{M_{M}}{M_{E}}{\left(\frac{R}{a}\right)}^{5}\sum_{q}\left[j_{Mq}\left(1-e^{2}\right)-j_{Wq}\sqrt{1-e^{2}}\right] & & \end{array}$$

$$\begin{array}{ccc} \times \left\{\begin{array}{c} -\frac{1}{2}{\left[U_{11q}+U_{22q}-2U_{33q}\right]}^{2}k_{20q}\sin\chi_{20q}\;\\ +3{\left[U_{23q}-U_{13q}\right]}^{2}k_{21q-}\sin\chi_{21q-}\;\\ -3{\left[U_{23q}+U_{13q}\right]}^{2}k_{21q+}\sin\chi_{21q+}\;\\ +\frac{3}{4}{\left[U_{11q}-U_{22q}+2U_{12q}\right]}^{2}k_{22q-}\sin\chi_{22q-}\;\\ -\frac{3}{4}{\left[U_{11q}-U_{22q}-2U_{12q}\right]}^{2}k_{22q+}\sin\chi_{22q+},\; \end{array}\right. & & \end{array}$$

and

(33) $$\begin{array}{ccc} \frac{di}{dt}=\frac{nS_{E}}{4\sin i\sqrt{1-e^{2}}}\frac{M_{M}}{M_{E}}{\left(\frac{R}{a}\right)}^{5}\sum_{q}\left[j_{Wq}\cos i-j_{\Omega q}\right] & & \end{array}$$

$$\begin{array}{ccc} \times \left\{\begin{array}{c} -\frac{1}{2}{\left[U_{11q}+U_{22q}-2U_{33q}\right]}^{2}k_{20q}\sin\chi_{20q}\;\\ +3{\left[U_{23q}-U_{13q}\right]}^{2}k_{21q-}\sin\chi_{21q-}\;\\ -3{\left[U_{23q}+U_{13q}\right]}^{2}k_{21q+}\sin\chi_{21q+}\;\\ +\frac{3}{4}{\left[U_{11q}-U_{22q}+2U_{12q}\right]}^{2}k_{22q-}\sin\chi_{22q-}\;\\ -\frac{3}{4}{\left[U_{11q}-U_{22q}-2U_{12q}\right]}^{2}k_{22q+}\sin\chi_{22q+},\; \end{array}\right. & & \end{array}$$

where $S_{E}$ is defined by

(34) $$S_{E}=1+\frac{{n\prime }^{2}}{2n^{2}}.$$

Lunar mean motion n enters into equations for da/dt, de/dt, di/dt, $S_{A}$, $S_{E}$, and energy dissipation rates ${Dissipation}_{22}$, ${Dissipation}_{21}$, and ${Dissipation}_{20}$. We solve for n through an inversion of Kepler's third law, modified to account for the mean attraction of the Sun (J. G. Williams & Boggs, 2016), viz.

(35) $$G(M_{E}+M_{M})=n^{2}a^{3}\left(1+\frac{{n\prime }^{2}}{2n^{2}}\right).$$

As with $a^{\prime }$, $n^{\prime }$ changes very slowly and is taken to be constant and equal to its present‐day value.

The precession rate of the equator, dψ/dt, also known as the “fundamental precession,” or “precession of the equinoxes,” has a present‐day period of about 26,000 years, and it contributes to Milankovitch cycles that perturb Earth's climate on time scales of order tens of thousands of years, for example, over ice‐age cycles. The precession rate will change over geological time due to changes in $\omega_{E}$, ε, and a, and is approximately given by

(36) $$\frac{d\psi }{dt}\approx \frac{3}{2}\frac{C(\omega_{E})-A(\omega_{E})}{C(\omega_{E})}\frac{\cos(\varepsilon )}{\omega_{E}}\left[\frac{GM_{M}}{a^{3}}\frac{1-\frac{3}{2}\sin^{2}i}{{\left(1-e^{2}\right)}^{1.5}}+\frac{GM_{S}}{{a\prime }^{3}}\frac{1}{{\left(1-{e\prime }^{2}\right)}^{1.5}}\right],$$

where the two terms on the right‐hand side are due to the Moon and Sun, respectively, $A\left(\omega_{E}\right)$ is the equatorial moment of an oblate Earth, and

(37) $$C\left(\omega_{E}\right)-A\left(\omega_{E}\right)=\left[0.655\times 10^{6}\omega_{E}^{2}k_{2f}\right]C\left(0\right).$$

As in Equation 16, $\omega_{E}$ must be given in radians/second. Using the present‐day values of $\omega_{E}$ and $k_{2f}$ in this expression, the present‐day value of $J_{2}$, the “second zonal harmonic,” due to the oblate Earth is given by

(38) $$J_{2}=\frac{C\left(\omega_{E}\right)-A\left(\omega_{E}\right)}{M_{E}R^{2}}=0.3299\times 0.00324=1.069\times 10^{-3}.$$

In the first approximation, the so‐called secular perturbation of the planets is a normal mode (eigenvector/eigenvalue) problem. For climatic effects of precession we want the perihelion direction with respect to the node of the equator plane on the ecliptic plane. Because our equinox direction dψ/dt moves in a retrograde direction and we define the rate positive, the rate for climate associated with perihelion direction is $g_{i}+d\psi /dt$, where the $g_{i}$ are the normal mode frequencies for the planetary eccentricities/longitudes of perihelia. The periods $P_{clim}$ are related to the period of the fundamental precession $P_{eqnx}=2\pi /\left[d\psi /dt\right]$ via:

(39) $$P_{clim}={\left[\frac{1}{P_{eqnx}}+\frac{1}{P_{NM}}\right]}^{-1},$$

where $P_{NM}$ are the normal mode periods corresponding to the $g_{i}$ values (Laskar et al., 2011, their Table 6). The four most important climate periods $P_{clim}$, corresponding to $p_{1}$, $p_{2}$, $p_{3}$, $p_{4}$ in Meyers and Malinverno (2018), are 23.7, 22.4, 19.0, and 19.1 kyr. An equation analogous to (39), using different notation, is found in Walker and Zahnle (1986, their Equation 9). The period of variation of Earth′s obliquity, currently about 41,000 years (depending on the precession constant used), will also undergo substantial changes over geological time scales.

### 5.2 Evolution due to Tides Within the Moon

Solid‐body tidal dissipation within the Moon also affects Earth‐Moon evolution. Because, at the present day, the Moon is tidally locked into synchronous rotation, the analogue of Earth′s large $M_{2}$ tide has zero frequency. The resulting deformation is static on human timescales. The Moon′s largest periodic tides are monthly, due to the eccentric orbit and inclination of the lunar equatorial plane to the orbital plane. Smaller components occur at one‐half month and other periods (J. G. Williams & Boggs, 2015). Due to the Moon′s synchronous rotation, energy dissipation within the Moon cannot affect “spin,” and instead yields a contraction of the lunar orbit (a reduction in the semi‐major axis a). Tides within the Moon also change the eccentricity e and inclination i of the lunar orbit. We modify Equations 35–37 from J. G. Williams et al. (2001) that account for the main effects of tides within the Moon at a period of one‐month to include the small contributions from other periods, viz.

(40) $$\begin{array}{cc} \frac{da}{dt} & =-3k_{2Moon}\frac{M_{E}}{M_{E}+M_{M}}\frac{M_{E}}{M_{M}}{\left(\frac{R_{M}}{a}\right)}^{5}na\;\\ & \times \left\{\begin{array}{c} e^{2}\left[7-\frac{17}{2}\sin^{2}\left(i+I\right)\right]\frac{1}{Q_{l}}\;\\ +\sin^{2}\left(i+I\right)\left[1-\frac{3}{4}\sin^{2}\left(i+I\right)-e^{2}\right]\frac{1}{Q_{F}}\;\\ +\frac{1}{2}e^{2}\sin^{2}\left(i+I\right)\left[29-\frac{67}{4}\sin^{2}\left(i+I\right)\right]\frac{1}{Q_{F+l}}\;\\ +\frac{1}{8}\sin^{4}\left(i+I\right)\left[(5-9e^{2})\frac{1}{Q_{2F}}+\frac{21}{8}e^{2}\frac{1}{Q_{2F-l}}+\frac{495}{8}e^{2}\frac{1}{Q_{2F+l}}\right]\;\\ +\frac{1}{32}\sin^{6}\left(i+I\right)\left[3\left(1-5e^{2}\right)\frac{1}{Q_{3F}}+\frac{1}{2}e^{2}\frac{1}{Q_{3F-l}}+49e^{2}\frac{1}{Q_{3F+l}}\right],\; \end{array}\right. \end{array}$$

(41) $$\begin{array}{cc} \frac{de}{dt} & =-\frac{3}{4}k_{2Moon}\frac{M_{E}}{M_{E}+M_{M}}\frac{M_{E}}{M_{M}}{\left(\frac{R_{M}}{a}\right)}^{5}ne\;\\ & \times \left\{\begin{array}{c} \left(1-e^{2}\right)\left[14-17\sin^{2}\left(i+I\right)\right]\frac{1}{Q_{l}}\;\\ -\frac{1}{2}\sin^{2}\left(i+I\right)\left[2-\frac{3}{2}\sin^{2}\left(i+I\right)-2e^{2}\right]\frac{1}{Q_{F}}\;\\ -\frac{1}{4}\sin^{2}\left(i+I\right)\left[10-\frac{19}{2}\sin^{2}\left(i+I\right)\right]\frac{1}{Q_{F-l}}\;\\ +\frac{1}{4}\sin^{2}\left(i+I\right)\left[58-\frac{67}{2}\sin^{2}\left(i+I\right)\right]\frac{1}{Q_{F+l}}\;\\ -\frac{5}{8}\sin^{4}\left(i+I\right)\frac{1}{Q_{2F}}\;\\ +\frac{3}{32}e\sin^{4}\left(i+I\right)\left[-7\frac{1}{Q_{2F-l}}+55\frac{1}{Q_{2F+l}}\right]\;\\ +\frac{1}{128}\sin^{6}\left(i+I\right)\left[-\frac{3}{2}\frac{1}{Q_{3F}}-\frac{1}{4}\frac{1}{Q_{3F-l}}+\frac{49}{4}\frac{1}{Q_{3F+l}}\right]\;\\ +\frac{1}{256}\sin^{4}\left(i+I\right)\left[-\frac{1}{Q_{4F}}-\frac{1}{8}\frac{1}{Q_{4F-l}}+\frac{49}{8}\frac{1}{Q_{4F+l}}\right],\; \end{array}\right. \end{array}$$

(42) $$\begin{array}{cc} \frac{di}{dt} & =-\frac{3}{2}k_{2Moon}\frac{M_{E}}{M_{E}+M_{M}}\frac{M_{E}}{M_{M}}{\left(\frac{R_{M}}{a}\right)}^{5}n\frac{1}{{\left(1-e^{2}\right)}^{\frac{1}{2}}}\cot i\;\sin^{2}\left(i+I\right)\\ & \times \left\{\begin{array}{c} \left[1-\frac{3}{4}\sin^{2}\left(i+I\right)-e^{2}\right]\frac{1}{Q_{F}}\;\\ +\frac{1}{8}e^{2}\left[10-\frac{19}{2}\sin^{2}\left(i+I\right)\right]\frac{1}{Q_{F-l}}\;\\ +\frac{1}{8}e^{2}\left[58-\frac{67}{2}\sin^{2}\left(i+I\right)\right]\frac{1}{Q_{F+l}}\;\\ +\frac{1}{8}\sin^{2}\left(i+I\right)\left[(5-21e^{2})\frac{1}{Q_{2F}}+\frac{21}{4}e^{2}\frac{1}{Q_{2F-l}}+\frac{165}{4}e^{2}\frac{1}{Q_{2F+l}}\right]\;\\ +\frac{3}{32}\sin^{4}\left(i+I\right)\left[(1-5e^{2})\frac{1}{Q_{3F}}+\frac{49}{4}e^{2}\frac{1}{Q_{3F+l}}+\frac{1}{4}e^{2}\frac{1}{Q_{3F-l}}\right]\;\\ +\frac{1}{128}\sin^{6}\left(i+I\right)\left[(1-5e^{2})\frac{1}{Q_{4F}}+\frac{49}{4}e^{2}\frac{1}{Q_{4F+l}}+\frac{1}{4}e^{2}\frac{1}{Q_{4F-l}}\right],\; \end{array}\right. \end{array}$$

where $R_{M}$ is the radius of the Moon, $k_{2Moon}$ is the gravitational potential Love number for the Moon (J. G. Williams & Boggs, 2015), and the tidal Q values for the Moon at various periods are listed in Table 3. The equations above are approximations, but one‐month tides dominate present‐day lunar dissipation (J. G. Williams et al., 2001) to within a few percent.

**Table 3:** Lunar Q Factor Values, Computed From Absorption Band Relations^a^

Lunar Q factor	Relevant periods	Value
$Q_{l}$	1 month	32.01
$Q_{F}$	1 month	32.01
$Q_{F-l}$	6.0 years	129.6
$Q_{F+l}$	$\frac{1}{2}$ month	38.11
$Q_{2F}$	$\frac{1}{2}$ month	38.11
$Q_{2F-l}$	1 month	32.01
$Q_{2F+l}$	$\frac{1}{3}$ month	41.16
$Q_{3F}$	$\frac{1}{3}$ month	41.16
$Q_{3F-l}$	$\frac{1}{2}$ month	38.11
$Q_{3F+l}$	$\frac{1}{4}$ month	42.68
$Q_{4F}$	$\frac{1}{4}$ month	42.68
$Q_{4F-l}$	$\frac{1}{3}$ month	41.16
$Q_{4F+l}$	$\frac{1}{5}$ month	43.45

^a^ See Figures 4 and 5 of J. G. Williams and Boggs (2015). The “l” and “F” arguments correspond to the “$\zeta_{q}$” arguments in J. G. Williams and Boggs (2015, see their Table 1 and associated text).

The inclination I of the lunar equatorial plane to the ecliptic plane is related to the lunar orbit inclination i via

(43) $$(3/2)n\frac{C_{Moon}-A_{Moon}}{C_{Moon}}\sin\left(i+I\right)\cos\left(i+I\right)+\left(3/8\right)n\gamma \sin\left(i+I\right)\left(1-\cos\left(i+I\right)\right)+\frac{d\Omega }{dt}\sin\left(I\right)=0,$$

(Ward, 1975); I is obtained from Equation 43 with an iterative solver. Here, $A_{Moon}$, $B_{Moon}$, and $C_{Moon}$ are the principal moments of inertia of the Moon with $A_{Moon}<B_{Moon}<C_{Moon}$, and $\gamma =\left(B_{Moon}-A_{Moon}\right)/C_{Moon}$. The ratio $(C_{Moon}-A_{Moon})/C_{Moon}$ can be written in terms of γ and $\beta =\left(C_{Moon}-A_{Moon}\right)/B_{Moon}$, numerical values of which can be found in J. G. Williams et al. (2014) and J. G. Williams et al. (2001), viz.

(44) $$\frac{C_{Moon}-A_{Moon}}{C_{Moon}}=\frac{\beta \left(1+\gamma \right)}{1+\beta }.$$

Equation 43 passes through resonance for values of n associated with values of a∼30−40R. Ward (1975) described this resonance passage, which is a change of Cassini state. In this paper, we have assumed that the lunar shape, and therefore the parameter values for α, β, and γ in Table 4, are fixed in time. This assumption may break down as the Moon and Earth draw closer and rotation rates increase (e.g., Le Bars et al., 2011, Supplementary Information). An alternative solution (J. G. Williams et al., 2001) for sin(I), and the effects of this alternative solution on our orbital dynamics results, are described in the Supporting Information S1 for this study.

**Table 4:** Parameters and Parameter Values Used in Integrations of the Earth‐Moon System Equations^a^ ^,^ ^b^ ^,^ ^c^

Parameter	Description	Value
Constant in time throughout integrations		
$a^{\prime }$	Semi‐major axis of Earth′s orbit around Sun	1.4960 $\times \;10^{11}$ m
$\alpha =\frac{C_{Moon}-B_{Moon}}{A_{Moon}}$	Ratio of principal moments of the Moon (see text)	$\frac{\beta -\gamma }{1-\beta \gamma }$
$\beta =\frac{C_{Moon}-A_{Moon}}{B_{Moon}}$	Ratio of principal moments of the Moon (see text)	6.31 $\times \;10^{-4}$
C(0)	Earth′s moment of inertia for zero spin rate	0.3299$M_{E}R^{2}$
$C_{Moon}/{C\prime }_{\mathrm{Moon}}$	Ratio of the whole Moon polar moment to the core polar moment	2,500
$C_{Moon}/\left[M_{M}R_{M}^{2}\right]$	Ratio of lunar polar moment of inertia to product of mass times square of radius	0.3929
$\frac{C_{Moon}-A_{Moon}}{C_{Moon}}$	Ratio of principal moments of the Moon (see text)	$\frac{\beta \left(1+\gamma \right)}{1+\beta }$
$e^{\prime }$	Eccentricity of Earth′s orbit around Sun	0.0316
G	Newton's gravitational constant	6.6738 $\times \;10^{-11}\;m^{3}\;{kg}^{-1}\;s^{-2}$
$\gamma =\frac{B_{Moon}-A_{Moon}}{C_{Moon}}$	Ratio of principal moments of the Moon (see text)	2.28 $\times \;10^{-4}$
$k_{2f}$	Earth fluid Love number for long‐term deformations	0.93
$k_{2Moon}$	Lunar gravitational potential Love number	0.02416
$M_{E}$	Mass of Earth	5.9726 $\times \;10^{24}$ kg
$M_{M}$	Mass of the Moon	7.3463 $\times \;10^{22}$ kg
$M_{S}$	Mass of the Sun	1.9885 $\times \;10^{30}$ kg
$n^{\prime }$	Sidereal mean motion of Sun	1.9910 $\times \;10^{-7}$ radians/second
R	Equatorial radius of Earth	6.378136 $\times \;10^{6}$ m
$R_{M}$	Lunar radius	1.738 $\times \;10^{6}$ m
$s_{f}$	Degree‐0 spherical distortion parameter	0.09
Evolving in time		
a	Semi‐major axis of lunar orbit around Earth	3.84399 $\times \;10^{8}$ m
e	Eccentricity of lunar orbit around Earth	0.0549
ε	Earth′s obliquity	0.4062 radians
i	Lunar inclination to ecliptic plane	0.0898 radians
I	Inclination of lunar equatorial plane to ecliptic plane	0.0274 radians
n	Sidereal mean motion of Moon	2.6617 $\times \;10^{-6}$ radians/second
$\omega_{E}$	Earth′s rotation rate	7.2921 $\times \;10^{-5}$ radians/second
ξ	Lunar fluid/mantle rotation coupling parameter	0.0226
$K_{Moon}/C_{Moon}$	Ratio of lunar core‐mantle torque factor to lunar moment of inertia about polar axis	9.67 $\times \;10^{-14}$ radians/second
κ	Dimensionless parameter depending on viscosity	7.48 $\times \;10^{-4}$
Auxiliary parameters that evolve in time		
$2\pi /\frac{d\bar{\omega }}{dt}$	Period of change of longitude of perigee	8.86 years
$2\pi /\frac{d\Omega }{dt}$	Nodal period	18.64 years
$2\pi /\frac{d\psi }{dt}$	Period of precession of the equinoxes	26.0 kiloyears

^a^ Entries separated into constant, time‐evolving, and auxiliary time‐evolving parameters, with present‐day values given.

^b^ Present‐day values of the auxiliary parameters and some other parameters (I, ξ, $K_{Moon}/C_{Moon}$, and κ) are computed from approximate formulae used in evolution equations.

^c^ α is used in Supporting Information S1.

The energy dissipation rate due to tides within the Moon is proportional to $\frac{da}{dt}$ because synchronous rotation causes nearly all dissipated energy to be extracted from the orbit rather than from lunar spin, viz.

(45) $$Dissipation_{Moon}=\frac{1}{2}\frac{GM_{E}M_{M}}{a^{2}}\frac{da}{dt}{|}_{Moon},$$

where $\frac{da}{dt}{|}_{Moon}$ is the $\frac{da}{dt}$ due to tides within the Moon, given by Equation 40. In the present‐day, because $\frac{da}{dt}{|}_{Moon}$ is ∼1.0% of the total $\frac{da}{dt}$, which is dominated by effects of tides on Earth, the energy dissipation rate due to tides within the Moon (∼1.2GW) is a small part (∼1.0%) of the total (Earth plus Moon) energy dissipation rate transferred into the orbit. In turn, the latter is ∼3% of the rate of total (Earth plus Moon) tidal energy dissipation. Thus the energy dissipation rate by tides within the Moon is about 0.03% of the total tidal energy dissipation rate. Because of the larger I near the Cassini state transition, the rates in Equations (40), (42) and 45 can be large near the change of spin state. Ward (1975) pointed out that the Moon will be heated during this transition. The amount of heating will depend on the tidal Q values, which could be larger for the early warmer Moon than in the present‐day.

### 5.3 Evolution due to Lunar Core‐Mantle Boundary Dissipation

Dissipation at the lunar core‐mantle boundary (CMB) also has a contracting influence on the semi‐major axis a, and affects the lunar inclination i. We follow J. G. Williams et al. (2001, Equations 81–84) to account for lunar CMB effects on orbit evolution:

(46) $$\frac{da}{dt}=-\frac{K_{Moon}}{C_{Moon}}\frac{1}{1+\xi^{2}}\frac{C_{Moon}}{M_{M}R_{M}^{2}}\left(1+\frac{M_{M}}{M_{E}}\right){\left(\frac{R_{M}}{a}\right)}^{2}2a\sin^{2}I,$$

(47) $$\frac{de}{dt}=0,$$

(48) $$\frac{di}{dt}=-\frac{K_{Moon}}{C_{Moon}}\frac{1}{1+\xi^{2}}\frac{C_{Moon}}{M_{M}R_{M}^{2}}\left(1+\frac{M_{M}}{M_{E}}\right){\left(\frac{R_{M}}{a}\right)}^{2}\frac{\sin^{2}I}{\sin i},$$

where $K_{Moon}$ allows for coupling between the fluid core and mantle (J. G. Williams et al., 2001, their Equation 40), $C_{Moon}$ is the Moon′s moment of inertia around the polar axis, and the parameter ξ allows for coupling between the fluid and mantle rotation (J. G. Williams et al., 2001, their Equation 46). ξ is given by

(49) $$\xi =-\frac{K_{Moon}}{C_{Moon}}\frac{C_{Moon}}{{C\prime }_{\mathrm{Moon}}}\frac{1}{\frac{d\Omega }{dt}}.$$

The torque at the core‐mantle interface is the product of $K_{moon}$ and the difference in the angular rotation rates of the core and mantle, where the latter is defined by

(50) $$\Delta {\vec{\omega }}_{CMB}={\vec{\omega }}_{core}-{\vec{\omega }}_{mantle}.$$

The torque on the mantle depends on the vector difference $\Delta {\vec{\omega }}_{CMB}$, and the torque on the core has the opposite sign. However, with small values of ξ, ${\vec{\omega }}_{core}$ is expected to point nearer the ecliptic pole than ${\vec{\omega }}_{mantle}$ even when sinI changes sign near the Cassini state resonance. Thus, we can use the magnitude $\Delta \omega_{CMB}$ of $\Delta {\vec{\omega }}_{CMB}$ in the definition of $K_{Moon}/C_{Moon}$, updated from J. G. Williams et al. (2001, Equation 55):

(51) $$\frac{K_{Moon}}{C_{Moon}}=\frac{45}{32}\pi \frac{{C\prime }_{\mathrm{Moon}}}{C_{Moon}}\kappa \Delta \omega_{CMB},$$

where $C_{Moon}/C_{Moon}^{\prime }$ is the ratio of whole Moon polar moment to core polar moment. The dimensionless parameter κ is defined in J. G. Williams et al. (2001, Equation 58); a simplified equation, under the assumption of a core with 330 km radius and a kinematic viscosity of 0.01 ${cm}^{2}\;s^{-1}$, is

(52) $$\sqrt{\kappa }=\frac{0.4}{14.631-\ln\left(1+\xi^{2}\right)}.$$

The equation for $\Delta \omega_{CMB}$ is

(53) $$\Delta \omega_{CMB}=\frac{\frac{dF}{dt}|\sin I|}{{\left(1+\xi^{2}\right)}^{1/2}},$$

where dF/dt=n−dΩ/dt, and the absolute value of sinI is taken because we are using the magnitude of $\Delta {\vec{\omega }}_{CMB}$. Values for the inter‐related parameters $K_{Moon}/C_{Moon}$, κ, and ξ are obtained with an iterative solver.

The energy dissipation rate by the lunar CMB interaction is given in J. G. Williams et al. (2001, Equation 81b), viz.

(54) $$Dissipation_{CMB}=\frac{K_{Moon}}{C_{Moon}\left(1+\xi^{2}\right)}\frac{C_{Moon}}{M_{M}R_{M}^{2}}M_{M}R_{M}^{2}n^{2}\sin^{2}I.$$

At the present day, the energy dissipation rate associated with the lunar CMB interaction is much smaller than the dissipation rate due to tides within the rest of the Moon.

### 5.4 Parameter Values

Table 4 lists the present‐day values of key parameters used in the orbital dynamics equations, along with some important auxiliary parameters. As with the Q values in Table 3, parameters in the upper part of Table 4 are taken to be constant in time. Parameters in the lower parts of Table 4, on the other hand, evolve with time. For parameters that change over geological time, long‐term mean values at the present day, with shorter period variations averaged out, are given. As an example, the value of obliquity ε in Table 4 has the Milankovitch cycle variability removed (Laskar et al., 2004).

### 5.5 Present‐Day *k* sin *χ* Values, Dissipation Rates, and Evolution Rates

Present‐day Earth and ocean tide ksinχ values, for all 14 constituents used in our orbital dynamics model, are provided in Table 5. The Earth tide ksinχ values are taken from Petit and Luzum (2010). The ocean tide values for $M_{f}$, $M_{m}$, the four largest diurnal constituents, and the four largest semi‐diurnal constituents are taken from J. G. Williams and Boggs (2016, Table 6). The ocean tide ksinχ values for the diurnal Ω and L+F tides are assumed equal to those for the nearby $K_{1}$ and $O_{1}$ tides, respectively. Likewise, the ocean tide ksinχ values for the semi‐diurnal Ω and L+F tides are assumed equal to those for $K_{2}$ and $M_{2}$, respectively. The present‐day ocean tide ksinχ values can vary by up to about 30% across different constituents. The Earth tide ksinχ values are smaller than the ocean values, and vary by greater amounts, with the $K_{1}$ Earth tide ksinχ value changing sign. Present‐day dissipation rates, computed from Equations (20), (22) and 23, are also provided in Table 5. The dissipation rates of the 14 constituents listed in the Table vary by almost five orders of magnitude from $M_{2}$ to $M_{m}$.

**Table 5:** Present‐Day Values of Earth Tide ksinχ, Ocean Tide ksinχ, and Dissipation (Earth + Ocean Tides) for the 14 Tidal Constituents Used in Our Orbital Dynamics Model

Constituent	Earth tide ksinχ	Ocean tide ksinχ	Dissipation (W)
Long‐period tides			
$M_{f}$	0.0021	0.0098	5.05e+08
$M_{m}$	0.0024	0.0066	5.58e+07
Diurnal tides			
$K_{1}$	−0.0012	0.0162	2.69e+11
$O_{1}$	0.0014	0.0220	1.96e+11
$P_{1}$	0.0007	0.0161	3.26e+10
$Q_{1}$	0.0014	0.0226	7.14e+09
Ω ($K_{1}$ nodal)	−0.0015	0.0162	4.92e+09
L+F ($O_{1}$ nodal)	0.0014	0.0220	7.11e+09
Semi‐diurnal tides			
$K_{2}$	0.0013	0.0175	3.11e+10
$M_{2}$	0.0013	0.0237	2.57e+12
$S_{2}$	0.0013	0.0171	4.22e+11
$N_{2}$	0.0013	0.0308	1.20e+11
Ω ($K_{2}$ nodal)	0.0013	0.0175	2.80e+09
L+F ($M_{2}$ nodal)	0.0013	0.0237	3.52e+09

The present‐day secular change rates in a, e, i, ε, and $\omega_{E}$, due to tides on Earth, computed from the equations used in our orbital dynamics model, are given in Table 6. Our rates are computed using approximations in the equations for the Cartesian factors U, thus introducing an error of a few percent relative to the values in J. G. Williams and Boggs (2016, Tables 7 and 8), which were computed from more precise numerical values of the Cartesian factors U. We consider this few percent level of agreement to be adequate for our purposes. The present‐day time rates of change of a, e, and i due to tidal and core‐mantle boundary (CMB) dissipation within the Moon (also given in Table 6) agree with results computed from the “DE440/DE441” model of lunar and planetary ephemerides (Park et al., 2021; J. G. Williams et al., 2021), to within 20% or less.

**Table 6:** Present‐Day Secular Rates of Change of Earth Rotation Rate $\omega_{E}$, Obliquity ε, and Earth‐Moon Orbital Parameters a (Semi‐Major Axis), e (Eccentricity), and i (Inclination), due to Tides on Earth, Tides Within the Moon, and Lunar Core‐Mantle Boundary Effects (CMB)^a^ ^,^ ^b^

Constituent	$\frac{da}{dt}\left(\frac{mm}{year}\right)$	$\frac{de}{dt}\left(\frac{10^{-12}}{year}\right)$	$\frac{di}{dt}\left(\frac{\mu as}{year}\right)$	$\frac{d\varepsilon }{dt}\left(\frac{\mu as}{year}\right)$	$\frac{d\omega_{E}}{dt}\left(\frac{\prime \prime }{century^{2}}\right)$
Long‐period tides					
$M_{f}$	−0.161	0.0057	0.0019	−0.528	0
$M_{m}$	−0.0178	−0.416	0	0	0
Diurnal tides					
$K_{1}$	0	0	0	−9.51	−93.7
$O_{1}$	4.98	−0.176	−0.0596	8.83	−73.8
$P_{1}$	0	0	0	1.36	−11.4
$Q_{1}$	0.283	2.20	−0.0023	0.335	−2.80
Ω ($K_{1}$ nodal)	0	0	−0.172	0.0154	−1.72
L+F ($O_{1}$ nodal)	0.181	−0.0064	0.266	0.0241	−2.68
Semi‐diurnal tides					
$K_{2}$	0	0	0	−1.10	−10.9
$M_{2}$	31.3	−1.11	−0.375	8.37	−930
$S_{2}$	0	0	0	1.32	−147
$N_{2}$	2.23	17.4	−0.0178	0.397	−44.2
Ω ($K_{2}$ nodal)	0	0	−0.0489	−0.0452	−0.978
L+F ($M_{2}$ nodal)	0.0429	−0.0015	0.0633	−0.0590	−1.27
Tides within the Moon^c^					
–	−0.387 (−0.471)	−5.49 (−6.9)	−0.459 (−0.51)		
Lunar core‐mantle boundary (CMB)^c^					
–	−0.0143 (−0.015)	0 (0)	−0.0429 (−0.04)		

^a^ Shown are results for 14 constituents of tides on Earth, as included in our orbital dynamics model.

^b^ Effects of lunar tides and lunar core‐mantle boundary (CMB) computed respectively from Equations (40), (41), (42) and (46), (47), (48), and parameter values used in this study.

^c^ DE440 values are given in parentheses.

### 5.6 Time‐Stepping Methods

We have presented a set of ordinary differential equations (ODEs) for the temporal evolution of the Earth‐Moon system. The time rates of change of the Earth rotation rate $\omega_{E}$, obliquity ε, and Earth‐Moon orbital parameters a, e, and i, due to tides on the Earth, are governed by Equations 15, (25), (26), (27) and (31), (32), (33). The time rates of change for a, e, and i due to tides within the Moon are given by Equations (40), (41), (42), while Equations (46), (47), (48) represent the time rates of change of a, e, and i due to dissipation at the lunar core‐mantle boundary.

**Figure 5:**
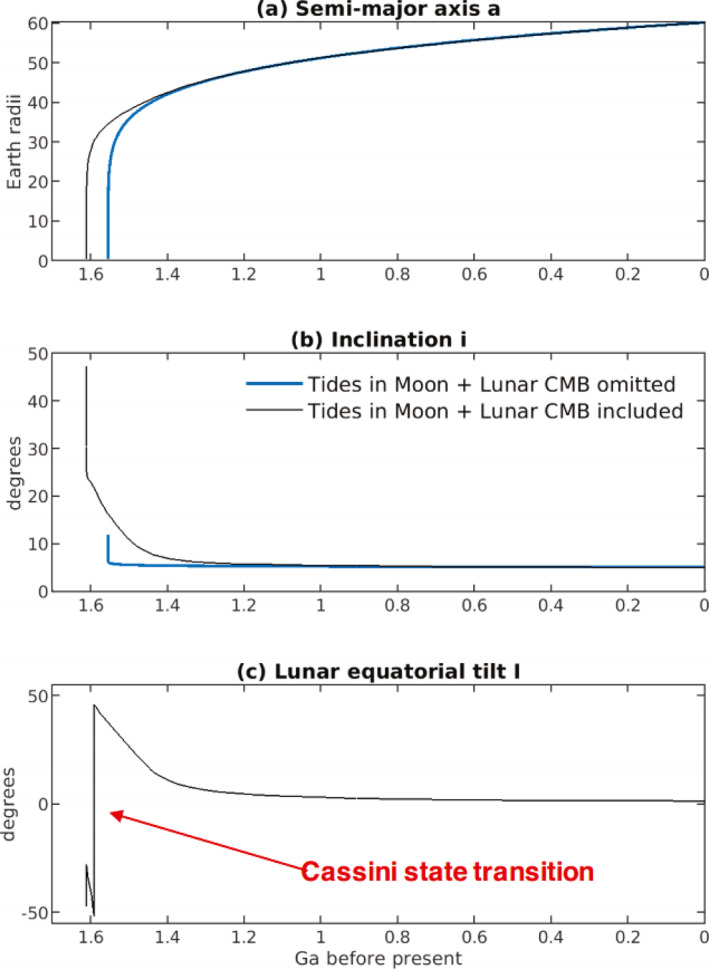
(a) Semi‐major axis a and (b) inclination i in simulations with constant ocean tide ksinχ values taken from present‐day conditions. Solutions that both omit (thick blue curves) and include (thin black curves) tidal and core‐mantle boundary (CMB) dissipation within the Moon are shown. (c) Lunar equatorial tilt I, in the solution that includes tidal and CMB dissipation within the Moon.

**Figure 6:**
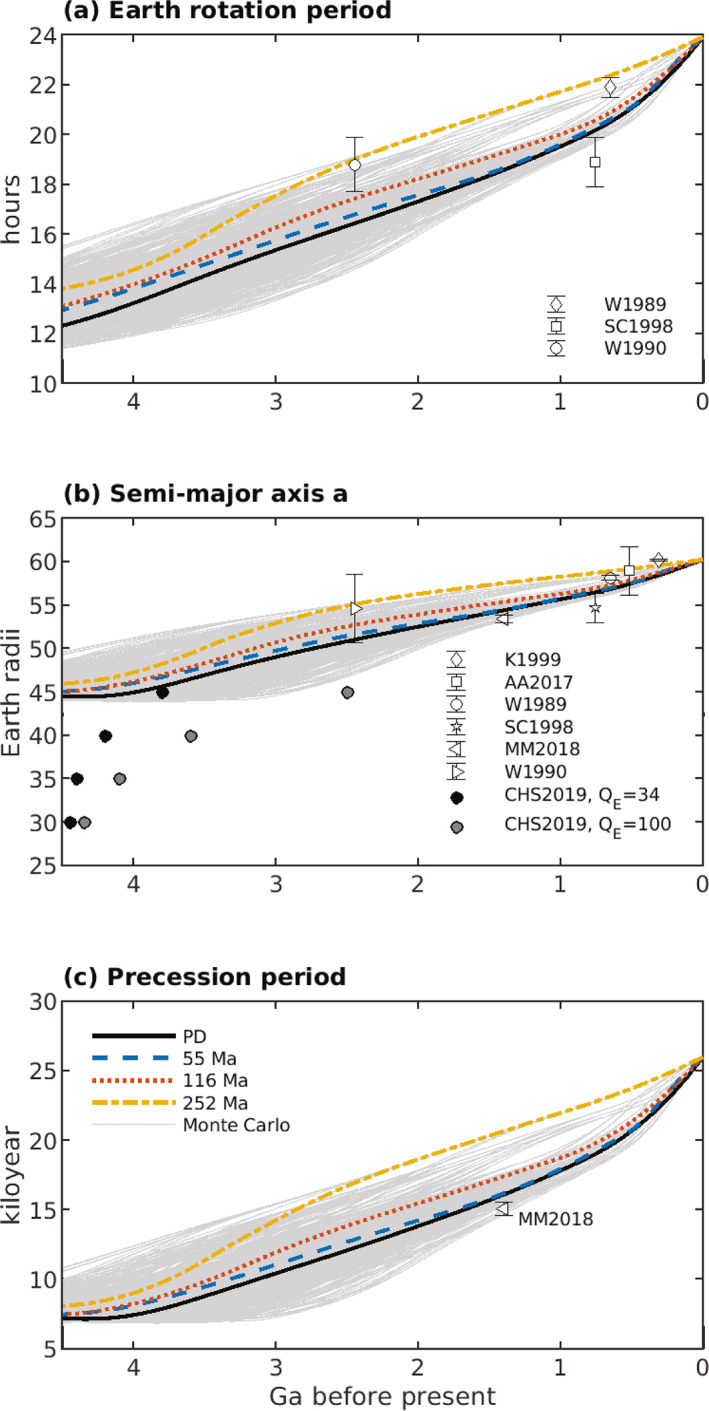
Modeled (a) Earth′s rotation period $2\pi /\omega_{E}$, (b) semi‐major axis a, and (c) Milankovitch precession period 2π/[dψ/dt], where dψ/dt, the “fundamental precession,” or “precession of the equinoxes,” is given by Equation 36 over 4.5 Ga. Four of the orbital dynamics results (see legend in (c)) are obtained with ocean tide ksinχ values taken from simulations that assume fixed basin geometries (see Figure 1) over time. The 1,000 Monte Carlo realizations (gray curves) employ lists in which, for each value of T (see Section 4), the ocean tide ksinχ value is chosen randomly from one of the four different paleogeographies. Tidal rhythmite estimates in (a–c) are tabulated in Table 7. The black and gray filled circles in (b) represent the results of simple “constant‐Q” forward models of early Earth‐Moon system tidal evolution as calculated by Ćuk et al. (2019, their Table 1) with two different values (34 and 100, respectively) of the early Earth tidal quality factor $Q_{E}$.

**Figure 7:**
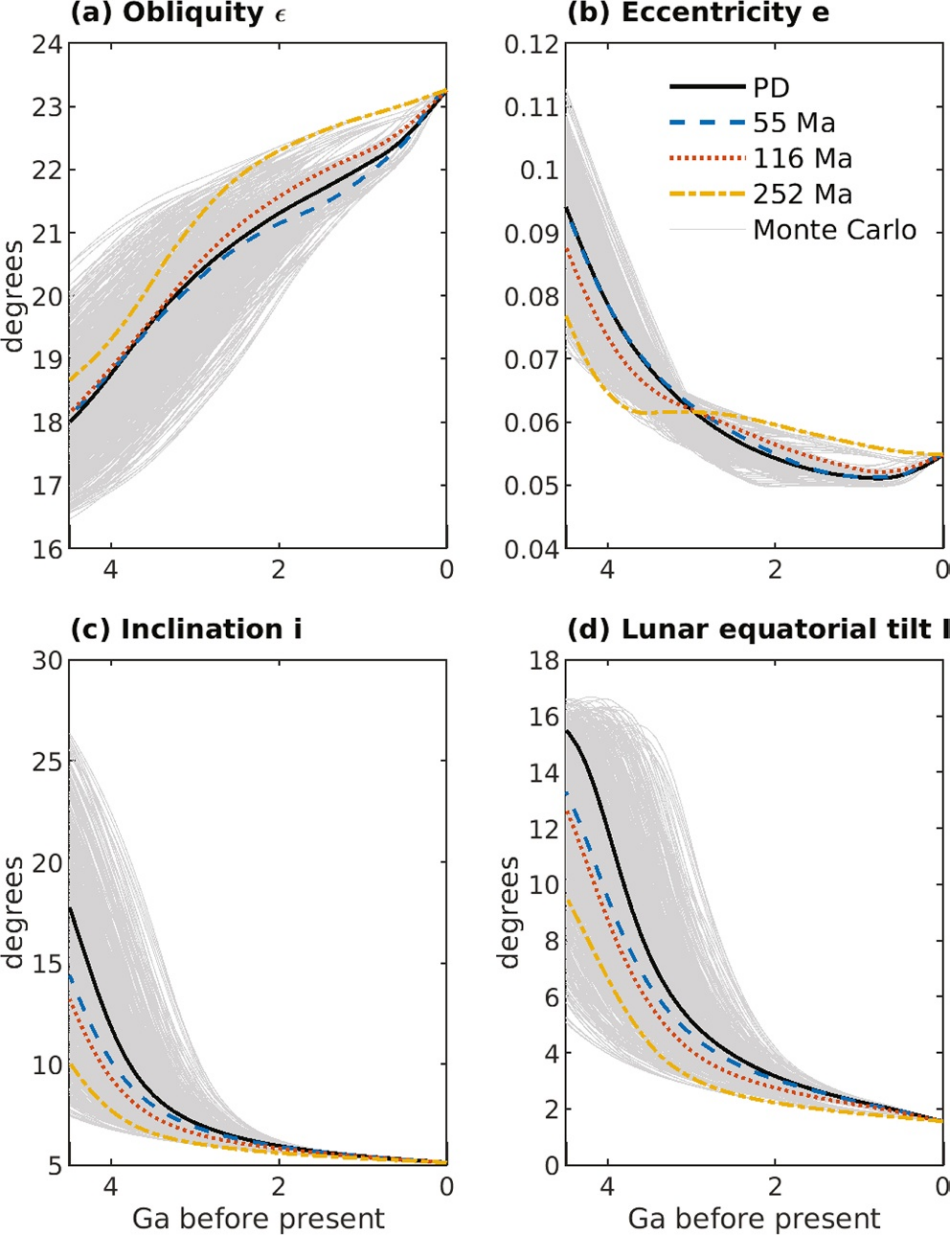
As in Figure 6, but for (a) mean obliquity ε (with Milankovitch variations removed), (b) eccentricity e, (c) inclination i, and (d) lunar equatorial tilt I.

**Figure 8:**
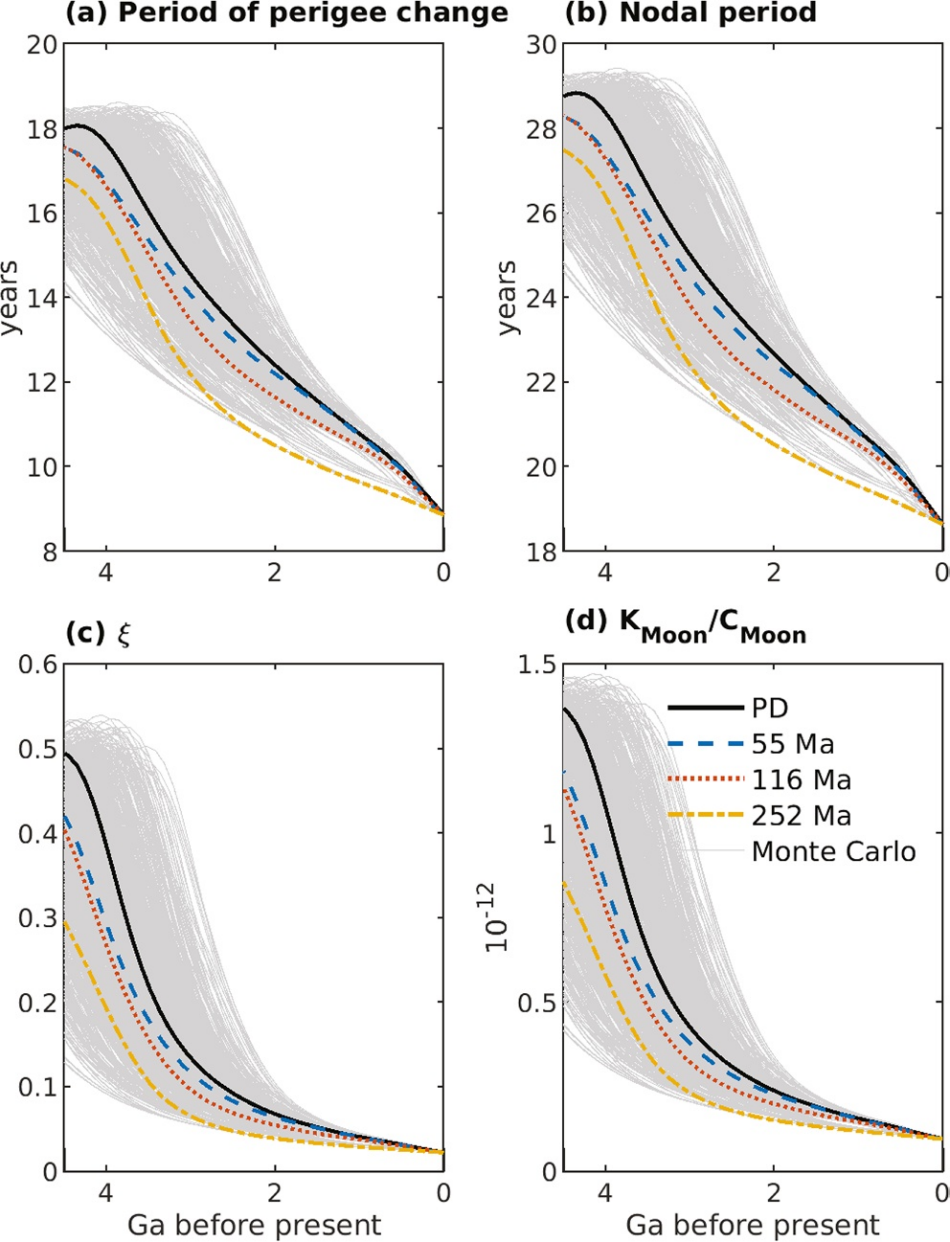
As in Figure 6, but for (a) period $2\pi /\left[d\bar{\omega }/dt\right]$ associated with the rate of change of longitude of perigee (Equation 4), (b) nodal period −2π/[dΩ/dt] (Equation 5), (c) ξ, the lunar core‐mantle boundary parameter (Equation 49), and (d) $K_{Moon}/C_{Moon}$ (Equation 51), another parameter in the lunar core‐mantle boundary equations (Section 5.3). Note that dΩ/dt is negative.

**Figure 9:**
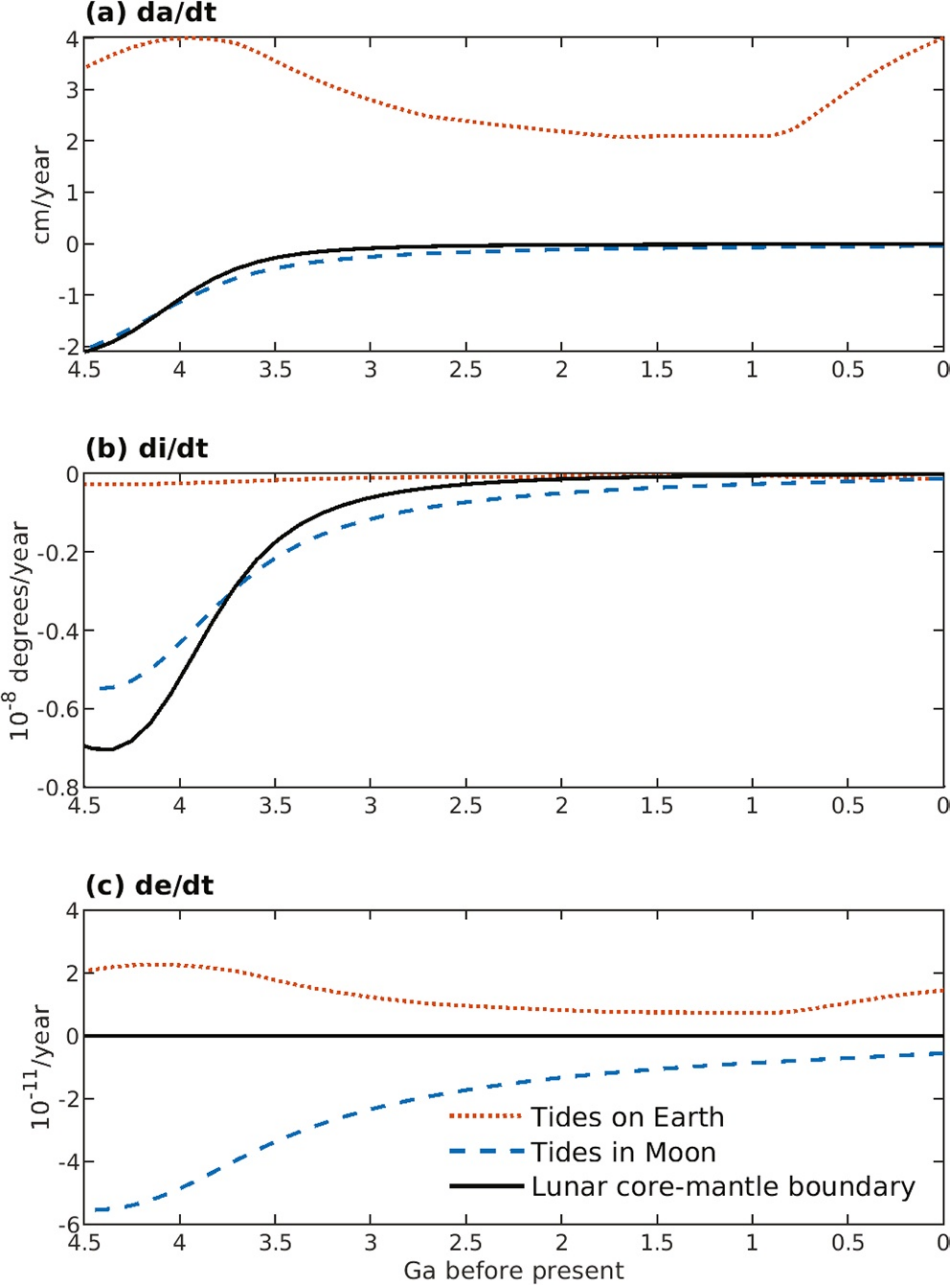
Time derivative d/dt of (a) semi‐major axis a, (b) inclination i, and (c) eccentricity e, in the orbital dynamics simulation that employs ksinχ values from the ocean tide model and fixed present‐day (PD) geometry. Separate time derivative terms associated with tides on Earth, tides within the Moon, and the lunar core‐mantle boundary (CMB) are shown in all three frames.

**Figure 10:**
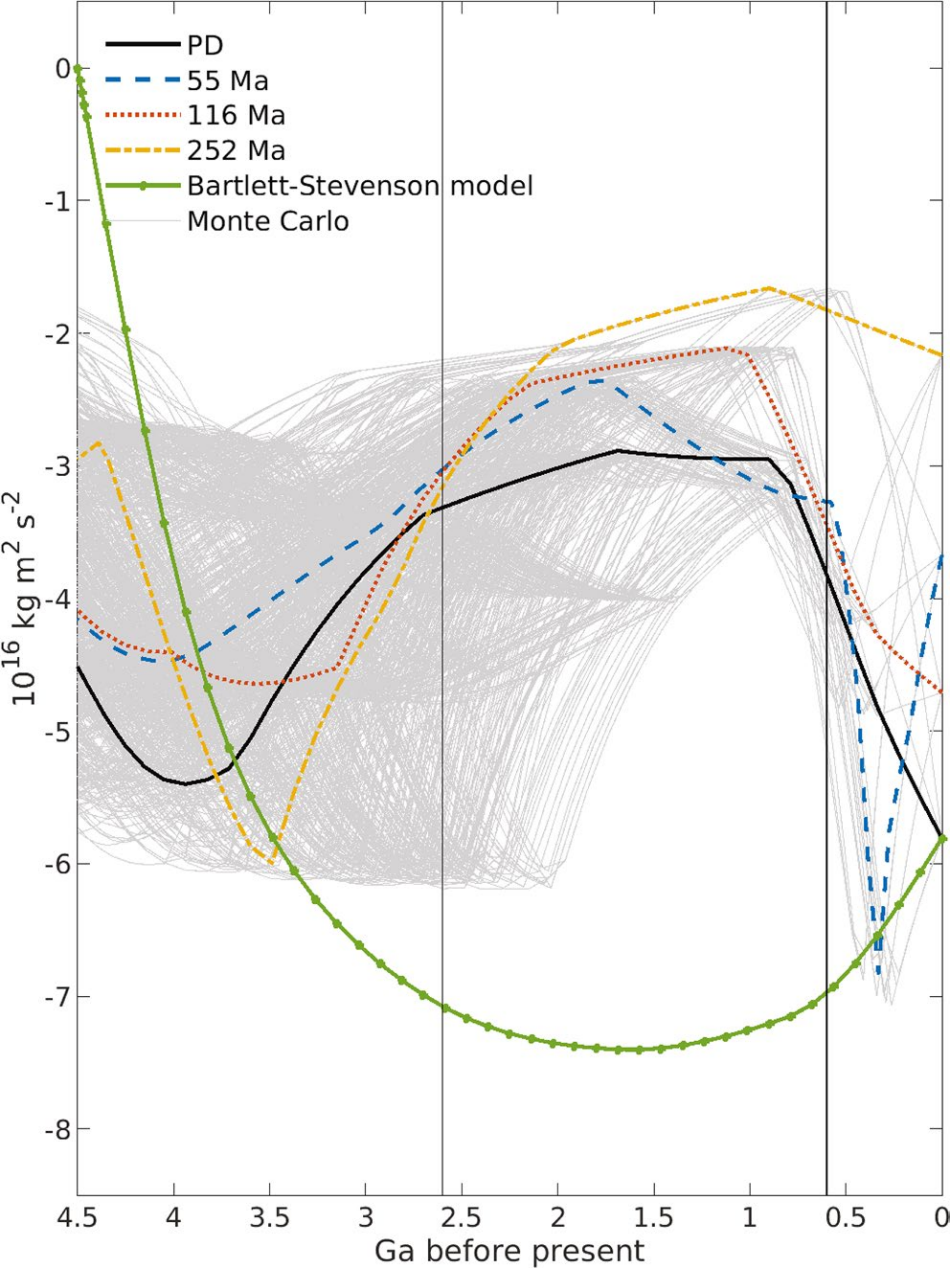
As in Figure 6, but for torques about the pole from tides raised on Earth. Torques from the simplified lunar torque model employed in Bartlett and Stevenson (2016), with semi‐major axis values taken from our “PD” fixed geometry simulation, are also given. Thin vertical black lines denote the approximate boundaries of the 0.6–2.6 Ga period of potential Earth rotation rate stabilization explored by Bartlett and Stevenson (2016).

**Figure 11:**
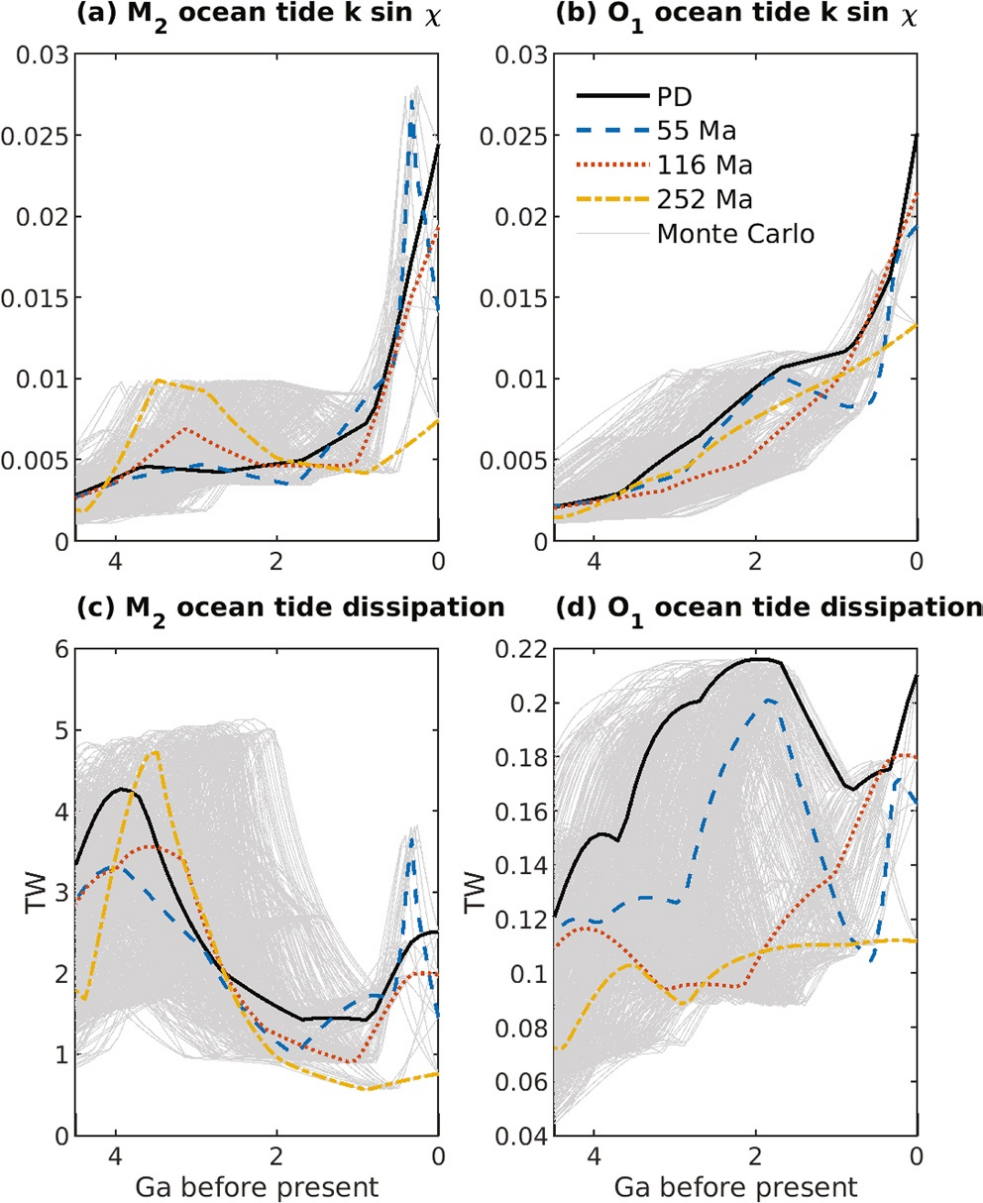
As in Figure 6, but for ksinχ values (a–b) and ocean tide energy dissipation rates (c–d) of (a, c) $M_{2}$ and (b, d) $O_{1}$.

The orbital dynamics model is time‐stepped using Matlab ODE solver packages. Orbital dynamics solutions obtained using ocean tide model results (Figures 6, 7, 8, 9, 10, 11) use the “ode45” package; solution robustness was checked through comparison with results from the “ode23” package. For these solutions trajectories are smooth and time steps are large, of order 100 million years. The solutions displayed in Figure 5 assume a constant value of ksinχ, change more rapidly, and employ the “ode23s” stiff ODE solver.

The orbital dynamics model takes Earth tide ksinχ values to be constant, and equal to their present‐day values (see Table 5). Tidal energy dissipation rates obtained in our ocean tide model simulations for the discrete set of rotation rates are converted into ksinχ values via Equations (20), (22) and 23, using present‐day values of a, n, $S_{A}$ and other constants, and the $\omega_{E}$ value applicable to the simulation in question (Table 2). As we integrate the Earth‐Moon system model, we perform a linear interpolation of ocean tide ksinχ values for the discrete set of simulated rotation rates, to particular values of $\omega_{E}$ that we obtain while time‐stepping the orbital dynamics model. Hence, $\omega_{E}$ serves as a conduit for passing results from a manageable number of ocean tide simulations, performed beforehand with simplifying assumptions described in Section 4.1, into the Earth‐Moon system model. In the Earth‐Moon system model, we assume that the $M_{2}$ ocean tide ksinχ values hold for the other semi‐diurnal constituents, and that the $O_{1}$ ocean tide ksinχ values hold for other diurnal constituents. We assume as well that the value of ksinχ depends only on Earth′s rotation rate, and not on semi‐major axis a; because a is a primary control on tidal forcing amplitudes, the latter point is a linearity assumption that we have successfully tested in a small number of cases.

In the present study, we do not simulate any long‐period constituents with our ocean tide model, and instead assume that present‐day ocean tide values of $k_{20q}\sin\chi_{20q}$ for $M_{f}$ and $M_{m}$, given in Table 5, hold over all time. Although this assumption is open to question, the long period tides dissipate less than 1% of the tidal energy so the effect is small.

The orbital dynamics model is run using ocean tide ksinχ values from all four ocean basin geometries considered separately. We also perform 1,000 Monte Carlo simulations of the orbital dynamics model. In each of these Monte Carlo trajectories, for every discrete value of T (Section 4) ranging from 6 to 24 hr in steps of 2 hr, we randomly select ksinχ values for $M_{2}$ and $O_{1}$ from ocean tide simulations done with that value of T and with one of our four basin geometries. In this manner, 1,000 lists of ocean tide ksinχ values, which sample the basin geometries randomly for each discrete Earth rotation period, are then used in our time‐stepping orbital dynamics model. The orbital dynamics model interpolates the ksinχ list at discrete rotation rates to the values of $\omega_{E}$ encountered during the time‐stepping. For the Monte Carlo simulations, we found that the T values in the lists should not become finer than 2 hr apart; otherwise, the rapid changes in ksinχ arising from the use of different geometries caused numerical problems in some of the integrations.

### 5.7 Summary of Orbital Dynamics Equations

Diurnal and semi‐diurnal tides on Earth extract energy from Earth′s rotation, causing it to slow. Part of the extracted energy is dissipated by tides, and part goes into expanding the semi‐major axis of the geocentric lunar orbit. For the zonal (2, 0) terrestrial tides, Earth′s rotation is unaffected and energy dissipated by tides is extracted from the orbit, yielding a reduced semi‐major axis. Energy dissipated by tides and CMB interactions within the Moon also comes from the orbit and contracts the semi‐major axis. The eccentricity of the lunar orbit increases from diurnal and semi‐diurnal terrestrial tides, but decreases from zonal terrestrial tides and tides within the Moon. At present, the sum of these influences causes the semi‐major axis and eccentricity of the lunar orbit to expand, but during evolution, reversal of both early de/dt and early da/dt is possible, as we will see later. The obliquity increases with time and the inclination decreases with time. Obliquity rate dε/dt has positive ($M_{2}$, $O_{1}$, $P_{1}$, and $S_{2}$) and negative ($K_{1}$, $K_{2}$, and $M_{f}$) contributions with a positive sum. A resonant $K_{1}$ tide could temporarily modify the sign of the obliquity rate.

## 6 Orbital Dynamics Model Results

### 6.1 Orbital Dynamics Results With Constant *k* sin *χ* Values

Backwards‐in‐time integration under the assumption of constant, present‐day ocean tide ksinχ values (taken from Table 5) yields a Gerstenkorn event (Gerstenkorn, 1955, 1967, 1969); a collision between Earth and Moon (a values reaching the Roche limit) at about 1.6 Ga before present (Figure 5a), with a rapidly increasing lunar orbit inclination i (Figure 5b). Inclusion of tidal and CMB dissipation within the Moon, which counteract the effects of terrestrial tides on the evolution of the semi‐major axis, pushes the Gerstenkorn event further back in time by about 58 Ma (compare two curves in Figure 5a). In the backwards trajectories, a rapid increase in inclination is seen earlier in the solution with tidal and CMB dissipation within the Moon included, due to the large effects that Moon tides have on inclination. At an a value of about 30.3 Earth radii, the lunar equatorial tilt I undergoes a Cassini state transition (Ward, 1975)—a rapid change in sign—in the solution that includes tidal and CMB dissipation within the Moon (Figure 5c).

### 6.2 Orbital Dynamics Results Using Ocean Tide Model *k* sin *χ* Values

We now turn to Earth‐Moon system solutions that employ results from our ocean tide simulations. For these results, the values of ksinχ are not constant in time, due to the effects of Earth′s changing rotation rate and varying continental configurations (Section 4). Earth′s rotation period $2\pi /\omega_{E}$ (Figure 6a), semi‐major axis a (Figure 6b), and precession period 2π/[dψ/dt] (Figure 6c) all decrease as one traverses backwards in time. Geological proxy results are included for all three quantities plotted in Figure 6. Background on the proxy estimates is given in Table 7, and model/proxy comparisons will be discussed in Section 7.2. The semi‐major axis plot includes comparisons with the Ćuk et al. (2019) model of early Earth‐Moon evolution, to be discussed in Section 7.3. The period 2π/n of the lunar mean motion (not plotted) also decreases as one moves backward in time, via its coupling to a through Equation 35. Obliquity ε (Figure 7a) decreases in a backwards trajectory. Backwards to about 3 Ga, eccentricity e (Figure 7b) is relatively constant, while lunar orbit inclination i (Figure 7c) increases slowly. From about 3–4.5 Ga, both e and i increase rapidly as one traverses backwards in time. The lunar equatorial tilt I, like the lunar inclination i, increases dramatically in the backwards trajectory from about 3–4.5 Ga (Figure 7d). The inclination and lunar equatorial tilt display a wider spread in the 4.5 Ga values of the different Monte Carlo simulations than is seen in the other variables plotted in Figures 6 and 7. The period $2\pi /\left[d\bar{\omega }/dt\right]$ associated with the rate of change of longitude of perigee increases as one traverses backwards in time (Figure 8a), as does the nodal period −2π/[dΩ/dt] (Figure 8b). The lunar core‐mantle boundary parameters ξ (Figure 8c) and $K_{Moon}/C_{Moon}$ (Figure 8d) undergo order‐of‐magnitude increases in the backwards trajectories.

**Table 7:** Proxy Tidal Rhythmite Values for Earth′s Rotation Period $2\pi /\omega_{E}$, and Normalized Lunar Orbit Semi‐Major Axis a/R ^a^ ^,^ ^b^ ^,^ ^c^

Geological formation	Age (Ma)	Reference for age	$2\pi /\omega_{E}$ (hr)	a/R	Reference for $2\pi /\omega_{E}$ and/or a/R values
Brazil	313.5	Schmitz and Davydov (2012)		60.2 ± 0.16	Kvale et al. (1999)—K1999
Puncoviscana	515	Adams et al. (2008)		59.0 ± 2.82	de Azarevich and Azarevich (2017)—AA2017
Elatina	650	Rooney et al. (2015)	21.9 ± 0.4	58.2 ± 0.30	G. E. Williams (1989)—W1989
Big Cottonwood	755	Dehler et al. (2010)	18.9 ± 1	54.7 ± 1.71	Sonett and Chan (1998)—SC1998
Xiamaling	≈1,400	Zhang et al. (2015)		53.4 ± 0.20	Meyers and Malinverno (2018)—MM2018
Weelli Wolli	2,449	Barley et al. (1997)	18.8 ± 1.1	54.6 ± 3.92	G. E. Williams (1990)—W1990; see also Walker and Zahnle (1986)

^a^ Tidal proxy values, and abbreviations in right‐most column, are those used in Figures 6a and 6b.

^b^ Geological formations, ages, and references for age and Earth‐Moon system parameter values are also tabulated.

^c^ Meyers and Malinverno (2018)—MM2018—also estimated the precession periods $p_{1}$, $p_{2}$, $p_{3}$, and so on, from the Xiamaling formation. Their precession estimate “$p_{1}$” is converted to the fundamental precession period 2π/[dψ/dt], using Equation 39, before display in Figure 6c; a value of 15.11 ± 0.24 kyr is obtained.

The contributions from tides on Earth, tides within the Moon, and the lunar core‐mantle boundary to the time evolution d/dt of the semi‐major axis a, inclination i, and eccentricity e, in the fixed “PD” basin geometry case, are given in Figure 9. Consistent with inferences from Figure 5a, the effects of tidal and CMB dissipation within the Moon on the semi‐major axis a counteract the effects of tides on Earth (Figure 9a), especially from about 3–4.5 Ga before present. Tides within the Moon also counteract the tides on Earth in their effects on eccentricity e (Figure 9c). Tidal energy dissipation on Earth, within the Moon, and at the lunar CMB all increase lunar inclination as one integrates backwards in time. Over most of the backwards trajectory, the magnitude of the effect of tides and CMB dissipation within the Moon on inclination is much greater than the magnitude of the effects of tides on Earth (Figure 9b). The magnitude of the lunar CMB effects from about 3–4.5 Ga is magnified due to the large $K_{Moon}/C_{Moon}$ values seen over this period (Figure 8d).

The minimum, maximum, and mean 4.5 Ga endpoint values of the Earth‐Moon system parameters, computed across the Monte Carlo simulations, are given in Table 8. Ninety‐five percentage confidence intervals (CI), estimated via the Bias‐Corrected and Accelerated (BCa) bootstrapping method with re‐sampling (Efron, 1987), are also provided. $M_{2}$ ocean tide $Q_{E}$ values are computed from $M_{2}$ ocean tide ksinχ values assuming that $k_{2}$ = 0.302. Wide spreads in the Monte Carlo values, defined as a ratio of maximum to minimum values exceeding two, are seen in lunar inclination i, lunar equatorial tilt I, the lunar CMB parameters ξ and $K_{Moon}/C_{Moon}$, $M_{2}$ tidal dissipation, $M_{2}\;k\sin\chi$, and $M_{2}\;Q_{E}$. The ranges and CIs of the 4.5 Ga endpoint values of the Earth‐Moon system parameters represent uncertainty estimates for our backwards integrations. However, the orbital dynamics model is missing several important processes (Section 8.3). Thus, the mean values and uncertainty envelopes will be different in our future orbital dynamics results.

**Table 8:** Minimum, Mean (With 95% CI, Confidence Intervals), and Maximum 4.5 Ga Values From the Monte Carlo Simulations, for Earth‐Moon System Parameters^a^

Parameter	Units	Present‐day	4.5 Ga Min.	4.5 Ga Mean (95% CI)	4.5 Ga Max.
$2\pi /\omega_{E}$	hr	23.93	11.40	13.00 (12.95,13.05)	15.54
ε	° (degrees)	23.27	16.47	18.13 (18.08, 18.18)	20.51
a	R	60.27	44.00	45.62 (45.56, 45.67)	49.62
2π/n	days	27.32	17.03	17.98 (17.95, 18.01)	20.40
e	–	0.0549	0.0714	0.0915 (0.0910, 0.0920)	0.1128
i	° (degrees)	5.145	7.443	15.18 (14.92, 15.49)	26.39
I	° (degrees)	1.570	5.070	12.69 (12.51, 12.85)	16.61
$2\pi /\left[d\bar{\omega }/dt\right]$	years	8.864	14.16	17.12 (17.07, 17.16)	18.49
‐2π/[dΩ/dt]	years	18.64	24.61	27.82 (27.77, 27.86)	29.29
ξ	–	0.0226	0.1281	0.3913 (0.3853, 0.3967)	0.5320
$K_{Moon}/C_{Moon}$	$10^{-14}$ radians/second	9.67	41.46	111.5 (110.1, 113.0)	146.0
2π/[dψ/dt]	kiloyears	26.0	6.905	7.880 (7.842, 7.920)	10.86
$M_{2}$ dissipation	TW	2.514	0.8359	2.689 (2.635, 2.745)	4.773
$M_{2}$ ocean k sinχ	–	0.0237	0.0011	0.0027 (0.0026, 0.0028)	0.0060
$M_{2}$ ocean $Q_{E}$	–	12.74	50.53	111.8 (109.7, 114.0)	279.0

^a^ Present‐day values are provided in the third column.

## 7 Discussion of Orbital Dynamics Results

### 7.1 Conservation of Vertical Angular Momentum

Tian and Wisdom (2020) argue that the vertical angular momentum—the component of the Earth‐Moon system angular momentum that is perpendicular to Earth′s orbital plane—is quasi‐conserved over time, and therefore provides a strong constraint on orbital history. In their Earth‐Moon system model, vertical angular momentum is conserved to one part in a thousand over a values ranging from 5 to 50R. In our results, vertical angular momentum, given by

(55) $$L_{z}=C\left(\omega_{E}\right)\omega_{E}\cos\varepsilon +M_{M}a^{2}n\sqrt{1-e^{2}}\cos i,$$

is conserved to within 1%, in the constant ksinχ solutions of Figure 5, in the orbital dynamics solutions that employ our ocean tide model results (Figures 6, 7, 8, 9, 10, 11), and in solutions that employ our ocean tide model results but in which effects of tides in the Moon and the lunar CMB are omitted (not shown). The “Earth spin” component of $L_{z}$ (first term on the right‐hand side of Equation 55) varies by more than a factor of about two in our orbital dynamics solutions that employ ocean tide model results, and by a factor of about six in the constant ksinχ solutions. The conservation of the sum of the two components of $L_{z}$ thus represents a non‐trivial test of our orbital dynamics code. The 1% imbalance is larger than that in Tian and Wisdom’s (2020) work, likely due to their use of more sophisticated “symplectic map” ODE solvers.

### 7.2 Comparison With Geological Proxies

We compare our orbital dynamics results with selected geological proxy estimates made from tidal rhythmites. The error bars of Earth rotation period proxy estimates at 650, 755, and 2,449 Ma (Table 7), lie within the envelope of our Monte Carlo estimates (Figure 6a). The error bars of rhythmite semi‐major axis a estimates between 0.3 and 2.45 Ga also lie within our Monte Carlo envelopes (Figure 6b). As with their 1.4 Ga semi‐major axis result, the Meyers and Malinverno (2018) 1.4 Ga tidal rhythmite estimate of precession period lies at the edge of our Monte Carlo envelope of modeled precession period results (Figure 6c). The marginal agreement between our preliminary orbital dynamics model results and some of the proxy results suggests the need for further development of our orbital dynamics model (Section 8.3). In addition, finding new sedimentary archives of paleotidal information in the geological record, and better calibrating these proxies in modern environments with known tidal forcing, will improve the accuracy and temporal resolution of data constraining the history of the Earth‐Moon orbit.

### 7.3 Comparison With Early Earth‐Moon Evolution Model

A significant aspect of our results is that the lunar orbit semi‐major axis does not approach low values at ∼4.5 Ga, as would be predicted by lunar formation models. The values of a in our orbital dynamics simulations that employ ocean tide model results are never smaller than 44.0R, and these simulations do not experience the Cassini state transition in I as seen in Figure 5c. We compare our modeled a values with results from simplified forward models of the early Earth‐Moon system (as calculated by Ćuk et al., 2019, see their Table 1) in Figure 6b. The simplified forward models assume a constant quality factor, $Q_{E}$, for early Earth. Models with low $Q_{E}=34$ (i.e., high dissipation rates in Earth) approach our Monte Carlo envelopes at 3.8 Ga, but in models with higher $Q_{E}$ = 100 (i.e., lower dissipation in Earth) a evolves too slowly and does not converge with our Monte‐Carlo results. The 4.5 Ga endpoint $M_{2}$ ocean tide $Q_{E}$ values in our simulations are also large, ranging from 50.53 to 279.0 with a mean of 111.8 (Table 8). Large $Q_{E}$ values lead the simplified forward models to evolve too slowly and render our backward simulations unable to reach realistically small a values at 4.5 Ga. Strong dissipation mechanisms, apparently missing or underestimated in our current model, may be needed to reconcile our orbital dynamics results with those of lunar formation simulations.

If the tidal and CMB dissipation within the Moon are omitted from our orbital dynamics model, then the eccentricity e decreases as one evolves the system backwards in time (not shown), in contrast to the increase seen in Figure 7b, and the inclination i increases much more slowly (also not shown), such that the 4.5 Ga values are much lower than evident in Figure 7c. Thus, the inclusion of tidal and CMB dissipation within the Moon is required for predicting the lunar inclination and eccentricity, which represent critical constraints on lunar formation and early Earth‐Moon system models, back in time. Because the Moon does not closely approach the Earth in our current results, our inclination and eccentricity evolution rates likely represent underestimates.

### 7.4 Implications for Rotation Stabilization Hypothesis

We briefly examine an effect that may have tempered Earth‐Moon system evolution by stabilizing Earth rotation rate for a long period. The time evolution of the torques $d\left[C\left(\omega_{E}\right)\omega_{E}\right]/dt$ about the pole, raised by tides on Earth, are displayed in Figure 10. Torques from atmospheric thermal tides, which resonate at an Earth rotation period of about 21 hr, may have counteracted torques due to ocean tides, yielding a long period (∼2Ga) of relatively stable Earth rotation rate (Bartlett & Stevenson, 2016; Zahnle & Walker, 1987). Due to “…the absence of a reliable history of the lunar (oceanic) tide…,” Bartlett and Stevenson (2016) constructed a highly simplified model of the torques raised by lunar oceanic tides on Earth. Bartlett and Stevenson (2016) show that as long as the actual lunar oceanic tidal torques are not much larger in magnitude than this simple estimate, a long period of Earth rotation stabilization is possible. During the time of hypothesized Earth rotation rate stabilization, our modeled lunar oceanic tidal torques are smaller in magnitude (Figure 10) than the simplified lunar oceanic tidal torques predicted by Bartlett and Stevenson (2016). Our results therefore lend support to their argument for a possible stabilization of Earth′s rotation rate, but the inclusion of atmospheric tidal effects into our orbital dynamics model will allow for more definitive conclusions.

### 7.5 Climatic Implications

Our work has implications for understanding changes in Earth′s climate over geological time. Orbital dynamics impact obliquity, the location of solar perihelion with respect to the equinox, the value of solar eccentricity, and precession rate. We discuss the first and last of these here. Obliquity controls Earth′s seasons, but there are lesser influences from the other three effects. The 4.5 Ga obliquity ε in our model (Figure 7a; Table 8) is about 3–7° less than it is today. By way of comparison, the 3–7° secular changes in obliquity in our simulations exceed the present‐day range (2.4°) of Milankovitch‐cycle changes in obliquity, which exerted a significant control on the more recent ice age. The greater precession rates in deep time lie even farther from the chaotic obliquity zone (Laskar & Robutel, 1993, Figure 5c) than the present‐day precession rate, lending support to the hypothesis that the Moon serves to stabilize the obliquity, and therefore the climate variability, of Earth (Laskar et al., 1993; Lissauer et al., 2012).

Consistent with the ocean tide model results in Figure 4, trajectories of $M_{2}$ and $O_{1}$ ocean tide ksinχ values in the orbital dynamics model (Figures 11a and 11b) display a general tendency to decrease as one goes farther back in time, due to increases in Earth′s rotation rate. However, despite the smaller ksinχ values, the modeled $M_{2}$ ocean tide energy dissipation rates (Figure 11c) over the period 3–4.5 Ga are generally larger, with Monte Carlo minimum, mean, and maximum 4.5 Ga values of 0.8, 2.7, and 4.8 TW, than the present‐day value of about 2.5 TW. The high ocean tide energy dissipation rate in deep time, despite the reduction in ksinχ values, is due to the smaller values of semi‐major axis a, which increase astronomical tidal forcing.

Some papers in the literature state that the tidal energy dissipation rate over most of Earth′s history must have been lower than it is in the present‐day, because the assumption of a “constant present‐day tidal dissipation rate” leads to the 1.6 Ga Gerstenkorn event. This argument is not strictly correct. Instead, an assumption of constant “ksinχ” yields a Gerstenkorn event at 1.6 Ga. The tidal dissipation rate does not stay constant with constant ksinχ values, due to the factors of $1/a^{6}$ in the energy dissipation rate formulae.

It has been argued (e.g., W. Munk & Wunsch, 1998) that open‐ocean tidal dissipation, which ultimately leads to ocean mixing, exerts a strong control on the oceanic meridional overturning circulation, and hence Earth′s climate. Our results suggest that deep‐time ocean tidal dissipation may have been relatively strong due to the proximity of the Moon to the Earth. A careful examination of the partition between coastal versus open‐ocean tidal dissipation is merited in future work. Examination of the dissipation rates in basin geometries with less land mass, which may have characterized the early Earth (Johnson & Wing, 2020; Korenaga, 2013, 2018), also is warranted.

### 7.6 Implications for Exoplanets

It is essential to use Earth as a proxy for further understanding of coupled orbital and climate evolution, and habitability, on Earth‐like extrasolar exoplanets, for which we have limited information. Current observations of terrestrial exoplanets can only infer their mass and orbital period around their star (Winn & Fabrycky, 2015). Numerical models, based on equilibrium tides, are used to simulate potential orbital configurations. Numerical models also are used to simulate climatic conditions and habitability (Grimm et al., 2018; Kasting et al., 1993; Seager, 2013; Turbet et al., 2018). All of these modeling results are fraught with uncertainty. For instance, the results shown here and in other papers (Blackledge et al., 2020; Green et al., 2019) demonstrate that tidal dissipation is a strong function of planetary rotation rates and ocean basin geometries, neither of which will be well constrained on exoplanets. In turn, tides impact planetary rotation rate, obliquity, and orbital configuration, all of which are key controllers of habitability, and can yield tidal locking (synchronous rotation; e.g., Barnes, 2017).

The interplay between orbital parameters, obliquity, planetary rotation rate, atmospheric composition, and the amount of starlight received is complex (Del Genio et al., 2018; Way et al., 2016). For example, Venus is uninhabitable due to its proximity to the Sun, dense atmosphere, and very slow retrograde spin (Yang et al., 2014). Yet, in its distant past, it may have been habitable because it had daylengths of tens of days, an ocean, and a different atmospheric composition (Way et al., 2016). This habitable state may have been reached because solar tides in the putative ocean of early Venus rapidly spun it down from daylengths of a few days to tens of days (Green et al., 2019).

## 8 Summary and Outlook to Future Work

We have investigated the long‐term evolution (over 4.5 Ga) of ocean tides and the Earth‐Moon system, using ocean tide and orbital dynamics models that are both “high‐level,” that is, not idealized.

### 8.1 Summary of Ocean Tide Model Results

We use a global ocean tide model, with realistic ocean basin geometries from the present‐day (PD) and from three paleo‐reconstructions—55, 116, and 252 Ma. For each of the four geometries, we run separate simulations of the $M_{2}$ semi‐diurnal tide and $O_{1}$ diurnal tide with sidereal Earth rotation periods ranging from approximately 6 to 24 hr, in increments of approximately 2 hr. The ocean tide energy dissipation rate values are translated, via Equations (20), (22) and 23, to the ksinχ values employed in the orbital dynamics model. In line with earlier studies based upon idealized ocean tide models (Hansen, 1982; Kagan & Maslova, 1994; Webb, 1982), we find that increasing Earth′s rotation rate for a fixed basin geometry generally decreases tidal amplitudes and tidal energy dissipation rates.

### 8.2 Summary of Orbital Dynamics Model Results

The Earth‐Moon system model, or orbital dynamics model, time‐steps the evolution of Earth′s rotation rate $\omega_{E}$, obliquity ε of the Earth′s equator plane to the ecliptic plane, the semi‐major axis a and eccentricity e of the lunar orbit, and the inclination i of the lunar orbit relative to the ecliptic plane. The Earth‐Moon system model is integrated backwards in time to 4.5 Ga before present, the approximate formation time of the Earth‐Moon system. The orbital dynamics model includes effects of Earth tides, ocean tides, tides within the Moon, and lunar core‐mantle boundary effects. Earth′s rotation rate $\omega_{E}$ is of central importance, as it is simultaneously one of the controlling parameters of ocean tide ksinχ values, and a key time‐stepped variable in the orbital dynamics equations.

The orbital dynamics model employs 14 tidal constituents—the four largest semi‐diurnal tides ($M_{2}$, $S_{2}$, $N_{2}$, $K_{2}$), the four largest diurnal tides ($K_{1}$, $O_{1}$, $P_{1}$, $Q_{1}$), and the two largest long‐period tides ($M_{f}$, $M_{m}$), as well as the diurnal and semi‐diurnal node (Ω) and L+F terms, which have a disproportionately large effect on lunar inclination i. The ocean tide ksinχ values required at every time‐step of the orbital dynamics model are obtained through interpolation of the $M_{2}$ and $O_{1}$ ocean tide model ksinχ values obtained at different discrete rotation rates, to the rotation rate at that particular time‐step of the orbital model. Thus, $\omega_{E}$ serves as a conduit for bringing the ocean tide model simulation results into the Earth‐Moon system model. The $M_{2}$ and $O_{1}$ ocean tide ksinχ values are assumed to hold for all semi‐diurnal and diurnal constituents, respectively. The long‐period ocean tide values of ksinχ, and the Earth tide ksinχ values for all 14 constituents, are assumed to be constant in time, and equal to those of the present‐day.

We reproduce the Gerstenkorn event (Gerstenkorn, 1955, 1967, 1969)—a collision between Earth and Moon at 1.6 Ga—with ocean tide ksinχ values that are assumed constant in time and equal to present‐day values. The lunar inclination i takes on values as large as 47°, and the lunar equatorial tilt I changes Cassini state as predicted by Ward (1975).

We perform orbital dynamics simulations with ocean tide model results from all four paleogeometries. We also perform 1,000 Monte Carlo simulations, which roughly account for our uncertain knowledge of the history of ocean basins, and provide a spread of plausible 4.5 Ga endpoint values of the key Earth‐Moon system parameters. The widest ranges in the 4.5 Ga endpoint values are seen in lunar inclination i, lunar equatorial tilt I, lunar CMB parameters ξ and $K_{Moon}/C_{Moon}$, $M_{2}$ tidal dissipation, $M_{2}\;k\sin\chi$, and $M_{2}\;Q_{E}$. A drawback for our results is that the semi‐major axis at 4.5 Ga ranges from 44.0 to 49.6 Earth radii, much larger than the near‐zero values implied by lunar formation models. Therefore, there must be some physics, missing from our current model, that would bring the Earth and Moon closer at 4.5 Ga.

In Section 7, we connect our results to other threads in the literature, including: (a) a discussion of the vertical angular momentum (Tian & Wisdom, 2020); (b) comparison of our modeled results of Earth rotation rate, lunar orbit semi‐major axis, and precession rate to results derived from tidal rythmites; (c) comparison of our backwards‐in‐time trajectories of a to results from forward models of the early Earth‐Moon system; and (d) discussion of the hypothesis (Bartlett & Stevenson, 2016; Zahnle & Walker, 1987) that Earth′s rotation rate may have been stable for a long (∼2 billion year) period during the Precambrian. We also have briefly discussed climatic consequences of the work presented here. The Milankovitch precession period would have been much shorter in the distant past. Ocean tidal dissipation, which affects oceanic meridional overturning circulation and hence Earth′s climate, may have been relatively strong in the distant past (e.g., from about 3–4.5 Ga), despite low ksinχ values, due to a proximate Moon. Finally, our work has implications for exoplanets, because tidal dissipation affects (and is affected by) planetary rotation rate and orbital configurations, which in turn influence habitability.

### 8.3 Future Work

In future work, we will pursue several improvements to the results presented here.

We will perform ocean tide simulations with all 14 tidal constituents used in our orbital dynamics model, not just $M_{2}$ and $O_{1}$. Because ocean tide ksinχ values are sensitive to small changes in tidal forcing frequency (Table 5), the assumption made here, that $M_{2}\;k\sin\chi$ values hold for all semi‐diurnal constituents and $O_{1}\;k\sin\chi$ values hold for all diurnal constituents, will be revisited. We are likely to use NOAA Modular Ocean Model version 6 (MOM6) for this and other explorations given below. MOM6 was used in early stages of the present study and is currently undergoing important updates, including implementation of a fast SAL solver. More exploration of ocean tide ksinχ sensitivity to the reduced semi‐major axis a values during orbital evolution will also be undertaken.

We will explore paleogeometries that better sample the range of ocean geometry changes over geological time. Indeed, early Earth may not have had large continental land masses (Johnson & Wing, 2020; Korenaga, 2013, 2018). Furthermore, the reconstructed paleogeometries used here tend to be overly smooth, yielding overestimates of tides and tidal dissipation (Green et al., 2017). To minimize this smoothness, we may use recently developed paleogeography products (Merdith et al., 2021), or fractal models of basin geometries, as in Blackledge et al. (2020). We will also investigate sensitivities of tidal dissipation to uncertainties in the volume of water in the ocean, as in Byrne et al. (2020) and Green et al. (2020). Water volume is most important in resonant states (e.g., at present and around 420 Ma), and those states are short lived in a geological context, probably lasting around 20 Myr or so (e.g., Davies et al., 2020; Green et al., 2018). There is some evidence in the geological record for water on an early Earth (e.g., Cates & Mojzsis, 2007; O'Neil et al., 2012; Piani et al., 2020), but the details of emerging oceans and continents on the early Earth are still under debate. Another source of uncertainty is the history of large ice caps, which exert a strong control on mean sea level.

We will search for missing physics needed to bring the early Earth and Moon closer together; one candidate mechanism is Earth tide ksinχ values in early Earth. Earth tides may have been the predominant tidal energy dissipation mechanism on Earth during early Earth‐Moon evolution. Ross and Schubert (1989) argued that Earth tide dissipation in the early Earth may have been larger due to a reduction in viscosity arising from higher temperatures. Therefore, the assumption made here, of Earth tide ksinχ values fixed at their current‐day values, may need to be changed in future investigations. However, faster rotation rates on early Earth also may result in a predominantly elastic solid Earth response (Lau & Faul, 2019; Lau et al., 2015; Lau et al., 2017). Research on such trade‐offs and the resulting Earth tidal dissipation is underway. Related to mantle dissipation is friction arising from coupling between the core and the mantle (e.g., free core nutations; Greff‐Lefftz & Legros, 1999) and friction at fluid‐solid boundaries (e.g., Correia, 2006). We will build upon theories of length‐of‐day changes induced by this coupling (e.g., Buffett, 1996; Dumberry & Mound, 2010).

We will consider tides within the cooling Moon, which also may have changed over time. The dissipation at, and flattening of, the lunar CMB likely went through changes as rotation rate slowed, the inner core solidified, the lunar shape underwent changes (Le Bars et al., 2011), and the lunar dynamo weakened and died (Tikoo et al., 2017). In addition, the Moon′s rotation can become non‐synchronous during the Cassini‐state transition (Ćuk et al., 2016) and the Earth‐Moon system can undergo periods of chaotic behavior (see also Ćuk et al., 2019).

We will consider how to implement atmospheric thermal tide resonance (Bartlett & Stevenson, 2016; Zahnle & Walker, 1987), into our orbital dynamics equations.

Finally, we will examine how uncertainty envelopes of Earth‐Moon system parameters in simulations conducted with the above improvements match uncertainty ranges in both models that forward integrate the early Earth‐Moon system (e.g., Ćuk et al., 2016, 2019; Tian & Wisdom, 2020; Touma & Wisdom, 1994) and geological proxy results. The largest looming question is whether our Earth‐Moon system model can bring the Moon closer to Earth at 4.5 Ga, as expected from the standard paradigm of lunar formation.

## Supporting information

Supporting Information S1

## Data Availability

The MOM6 simulations used in early drafts of this study were carried out on the Flux supercomputer provided by the University of Michigan Advanced Research Computing Technical Services. Computational resources for the main ocean tide simulations used in this work were provided by the Vienna Scientific Cluster (VSC) and the NASA High‐End Computing (HEC) Program through the NASA Advanced Supercomputing (NAS) Division at Ames Research Center. Data sufficient to make Figures 1 and 4, 5, 6, 7, 8, 9, 10, 11, the 24 hr results in Figures 2 and 3, and Figures S1 and S2 are provided in Arbic and Schindelegger (2021). A subset of the computational code used in this paper is also provided in Arbic and Schindelegger (2021).
