# Supplementary material for: Long‐Term Earth‐Moon Evolution With High‐Level Orbit and Ocean Tide Models
Source: J Geophys Res Planets. 2021 Dec 1;126(12):e2021JE006875. doi: 10.1029/2021JE006875 (PMC9285098; doi:10.1029/2021JE006875)
Supplement: Supplementary file 1 — Supporting Information S1 [file JGRE-126-0-s001.pdf]

Supporting information for "Long-term Earth-Moon evolution with high-level orbit and ocean tide models"

Houraa Daher[1,2], Brian K. Arbic[3,4,5], James G. Williams[6], Joseph K. Ansong[3,7], Dale H. Boggs[6], Malte Müller[8], Michael Schindelegger[9], Jacqueline Austermann[10,11], Bruce D. Cornuelle[12], Eliana B. Crawford[3,13], Oliver B. Fringer[14], Harriet C.P. Lau[10,15], Simon J. Lock[16], Adam C. Maloof[17], Dimitris Menemenlis[6], Jerry X. Mitrovica[10], J.A. Mattias Green[18], Matthew Huber[19]

[1]Department of Climate and Space Sciences and Engineering, University of Michigan, Ann Arbor, MI, USA
[2]Rosenstiel School for Marine and Atmospheric Science, University of Miami, Miami, FL, USA
[3]Department of Earth and Environmental Sciences, University of Michigan, Ann Arbor, MI, USA
[4]Institut des Géosciences de L'Environnement (IGE), Grenoble, France
[5]Laboratoire des Etudes en Géophysique et Océanographie Spatiale (LEGOS), Toulouse, France
[6]Jet Propulsion Laboratory, California Institute of Technology, Pasadena, CA, USA
[7]Department of Mathematics, University of Ghana, Accra, Ghana
[8]Norwegian Meteorological Institute, Oslo, Norway
[9]Institute of Geodesy and Geoinformation, University of Bonn, Bonn, Germany
[10]Department of Earth and Planetary Sciences, Harvard University, Cambridge, MA, USA
[11]Department of Earth and Environmental Sciences, Columbia University, New York, NY, USA
[12]Scripps Institution of Oceanography, University of California, La Jolla, CA, USA
[13]Department of Physics, Kenyon College, Gambier, OH, USA
[14]Department of Civil and Environmental Engineering,Stanford University, Stanford, CA, USA
[15]Department of Earth and Planetary Sciences, University of California, Berkeley, CA, USA
[16]Division of Geological and Planetary Sciences, California Institute of Technology, Pasadena, CA, USA
[17]Department of Geosciences, Princeton University, Princeton, NJ, USA
[18]School of Ocean Sciences, Bangor University, Menai Bridge, UK
[19]Department of Earth, Atmospheric, and Planetary Sciences, Purdue University, West Lafayette, IN, USA

Alternative solution for lunar equatorial tilt

In the Daher et al. paper that this supporting information accompanies, the lunar equatorial tilt I is solved for using equation (43) of that paper, which is taken from Ward (1975). Here we present an alternative solution for I, taken from equation (52c) of Williams et al. (2001):

$$\sin I = \frac{G_t}{G_b}, \tag{1}$$

where

$$G_t = -3n^2 \sin i \cos(\sigma - \tau)(0.9865\beta + 0.0041\alpha + E), \tag{2}$$

$$G_b = 2.0002\frac{d\Omega}{dt}\omega_z + 3n^2(0.9754\beta + 0.0048\alpha + E) - 1.9982(\frac{d\Omega}{dt})^2 - 2\frac{dF}{dt}\frac{K_{Moon}}{C_{Moon}}\frac{\xi}{1+\xi^2}, \tag{3}$$

σ is a physical libration angle, and τ is the physical libration in longitude. Here

$$\alpha = \frac{C_{Moon} - B_{Moon}}{A_{Moon}} = \frac{\beta - \gamma}{1 - \beta\gamma}, \tag{4}$$

Corresponding author: Brian K. Arbic, arbic@umich.edu

$$E = \frac{k_{2Moon}\zeta}{3}, \qquad \zeta = \frac{M_M R_M^2}{C_{Moon}} \frac{M_E}{M_M} (\frac{R_M}{a})^3, \tag{5}$$

$$\frac{dF}{dt} = n - \frac{d\Omega}{dt}, \tag{6}$$

and ω_z, the body-referenced z-component of the Moon's angular velocity vector, is given by

$$\omega_z = \frac{d\Omega}{dt} \cos(I) + \frac{dF}{dt}. \tag{7}$$

We evaluate $\cos(\sigma - \tau)$ via Williams et al. (2001, equation 51):

$$\sin(\sigma - \tau) = -\frac{K_{Moon}}{C_{Moon}(1 + \xi^2)} \frac{2\frac{dF}{dt} \sin I \cos I}{3n^2 \sin i (0.9840\beta + 0.0059\alpha)}. \tag{8}$$

The non-integer coefficients depend on the average U_{ij} functions in Williams et al. (2001).

Because K_{Moon}/C_{Moon}, κ (see equation 52 in main text), ξ, and I are inter-related, they are obtained with an iterative solver. For some parameter values, small imaginary values crop up, and are omitted. Near and within the Cassini state transition, the solver is unable to find solutions for some values of a, in which case it assigns "NaN" (Not a Number) values to the parameters K_{Moon}/C_{Moon}, ξ, and I. For a small number of a values near or within the transition, parameter values can contain large imaginary values, or absolute values of I that exceed 90 degrees. In cases where the imaginary part of the solution of K_{Moon}/C_{Moon}, ξ, or I is 1% or more of the real part of the solution, or where $|I|$ exceeds 90 degrees, we assign values of all three parameters (K_{Moon}/C_{Moon}, ξ, and I) to "NaN".

We first compare the values of lunar equatorial tilt I using the Ward (1975) equation (equation 43, Daher et al.) versus the Williams et al. (2001) equation (1) in the case where the lunar inclination i is assumed to be fixed. We choose values of i=5.145°, the present-day value, and i=15.18°, the 4.5 Ga mean value of i from the Monte Carlo simulations (Table 8, Daher et al.), and plot I as a function of a (supporting information Figure 1). For both of these fixed i values, the values of I taken from the two solutions lie close together over the part of parameter space where $|I|$ is small. Both the Ward and Williams solutions display a Cassini state transition, near which the I values diverge from each other. In the Ward solutions, the transition takes place at about a=33.7R for the present-day i value of 5.145° and at about a=31.5R for the 4.5 Ga mean i value of 15.18°. In the Williams solutions for i=5.145°, there is a gap, between about 35.5 and 37.7 R, where only a small number of solutions for I that meet the criteria above (small imaginary values, assigned to non-NaN values, etc.) exists. Similarly, when i=15.18°, there is a gap between about 33.0 and 39.7 R with only a small number of solutions. A backwards integration of the orbital dynamics solutions in Daher et al. using (1) has the potential to fail, if a becomes small enough that the solutions for I fall into this gap, as may happen in some of our future simulations. For this reason, the solutions displayed in Daher et al. (Figures 5 to 11), employ the Ward (1975) (equation 43, Daher et al.).

In the Daher et al. orbital dynamics solutions that employ ocean tide model results, a never becomes small enough for a Cassini state transition to take place. Therefore, we are able to successfully employ the Williams et al. (2001) equation (1) in backwards 4.5 Ga integrations. We employed equation (1) in simulations that use the four

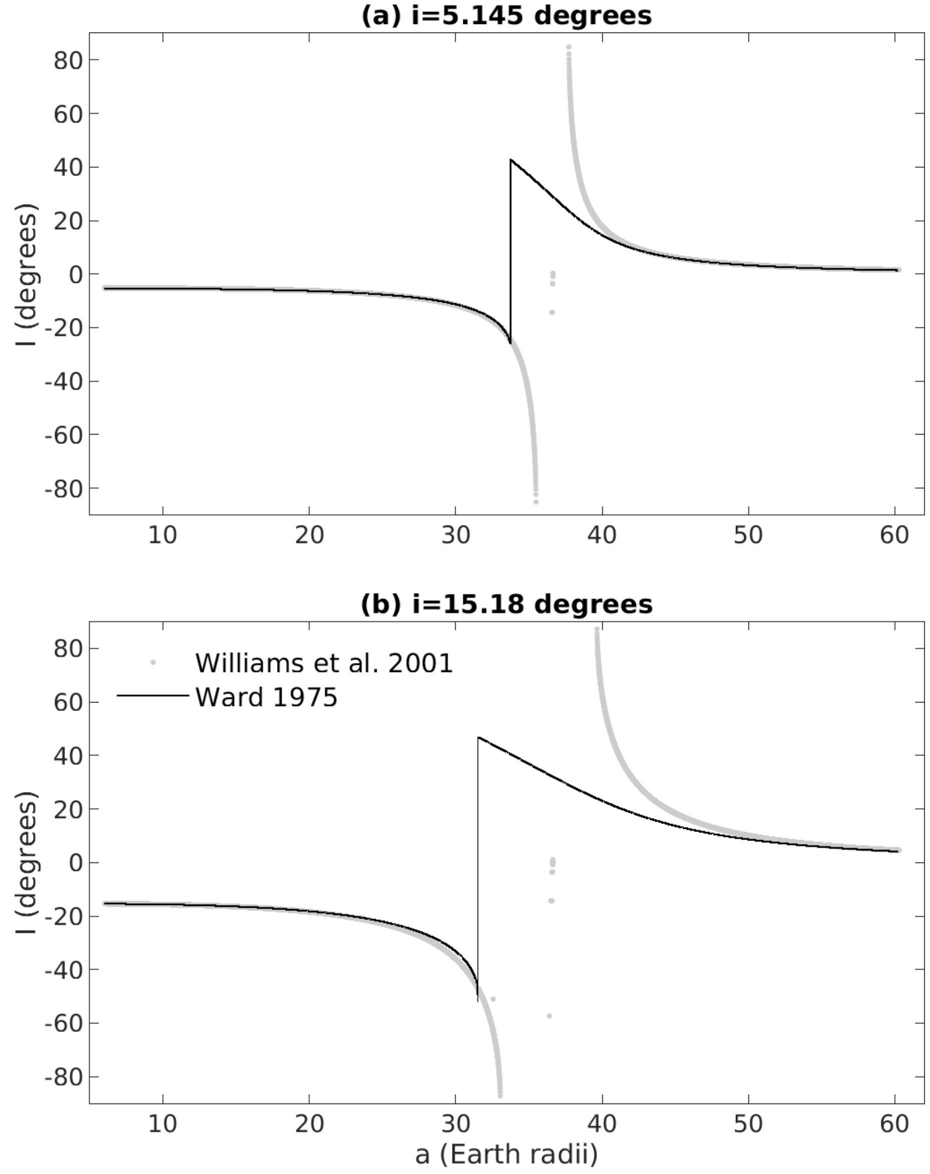

Figure 1. Lunar equatorial tilt I, plotted vs. semi-major axis a, computed from Williams et al. (2001) equation (1) and from Ward (1975) (equation 43 in Daher et al.), using (a) present-day i value of 5.145° and (b) the 4.5 Ga mean i value of 15.18° (Table 8 in Daher et al.). The curves are based upon 9001 values of semi-major axis a. The Williams et al. (2001) solutions are plotted as light-colored dots, which appear to be continuous over much of parameter space; small numbers of isolated solutions appear within the gap that covers the Cassini state transition.

fixed ocean basin geometries. Throughout the simulations employing the four basin geometries, the differences between solutions that employ the Williams et al. (2001) equation (1) vs. the Ward equation (43, in Daher et al.) are qualitatively similar. For brevity, we only display the solutions employing the PD geometry here. We plot Earth rotation period $\frac{2\pi}{\omega_E}$, obliquity ϵ, semi-major axis a, eccentricity e, lunar orbit inclination i, and lunar equatorial tilt I in supporting information Figure 2, for results that use the Williams et al. (2001) equation (1) vs. results obtained from the Ward (1975) solution (equation 43 in Daher et al.). The solutions for $\frac{2\pi}{\omega_E}$ and ϵ lie close together. The solutions for a and e display a small amount of divergence near the 4.5 Ga endpoint, and the solutions for i and particularly I show the greatest amount of divergence. This summarizes our alternative method for computing the lunar equatorial tilt I, and the effects that the alternative method have on the orbital dynamics solutions.

References

Ward, W. R. (1975). Past orientation of the lunar spin axis. *Science*, *189*, 377-379.

Williams, J. G., Boggs, D. H., Yoder, C. F., Ratcliff, J. T., & Dickey, J. O. (2001). Lunar rotational dissipation in solid body and molten core. *Journal of Geophysical Research*, *106*, 27933-27968. doi: 10.1029/2000JE001396

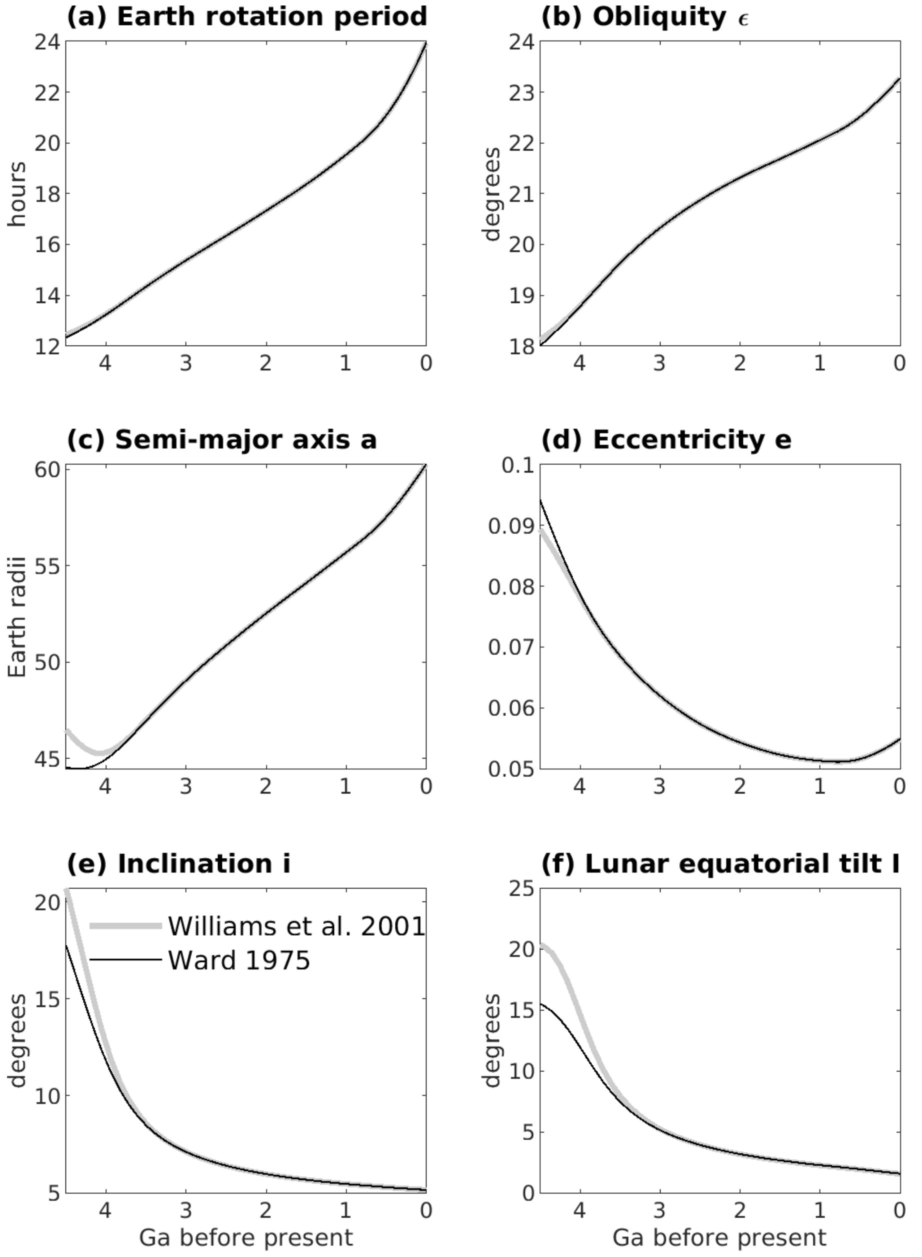

Figure 2. Orbital dynamics solutions employing the ocean tide results from fixed PD ocean basin geometry and the Williams et al. (2001) equation (1) vs. the Ward (1975) solution (equation 43 in Daher et al.) for lunar equatorial tilt I. Displayed are (a) Earth rotation period $\frac{2\pi}{\omega_E}$, (b) obliquity ε, (c) semi-major axis a, (d) eccentricity e, (e) lunar orbit inclination i, and (f) lunar equatorial tilt I.
